# The Fe–S cluster assembly protein IscU2 increases α-ketoglutarate catabolism and DNA 5mC to promote tumor growth

**DOI:** 10.1038/s41421-023-00558-8

**Published:** 2023-07-25

**Authors:** Xiaojun Ren, Jimei Yan, Qiongya Zhao, Xinzhu Bao, Xinyu Han, Chen Zheng, Yan Zhou, Lifang Chen, Bo Wang, Lina Yang, Xi Lin, Dandan Liu, Yuyan Lin, Min Li, Hezhi Fang, Zhimin Lu, Jianxin Lyu

**Affiliations:** 1 School of Laboratory Medicine and Bioengineering, Hangzhou Medical College, Hangzhou, Zhejiang China; 2 Laboratory Medicine Center, Department of Clinical Laboratory, Zhejiang Provincial People’s Hospital, Affiliated People’s Hospital, Hangzhou Medical College, Hangzhou, Zhejiang China; 3 Zhejiang Provincial Key Laboratory of Medical Genetics, Key Laboratory of Laboratory Medicine, Ministry of Education, School of Laboratory Medicine and Life Sciences, Wenzhou Medical University, Wenzhou, Zhejiang China; 4 Zhejiang Provincial Key Laboratory of Pancreatic Disease, The First Affiliated Hospital and Institute of Translational Medicine, Zhejiang University School of Medicine, Zhejiang University, Hangzhou, Zhejiang China; 5 Cancer Center, Zhejiang University, Hangzhou, Zhejiang China

**Keywords:** Cancer metabolism, Mechanisms of disease

## Abstract

IscU2 is a scaffold protein that is critical for the assembly of iron–sulfur (Fe–S) clusters and the functions of Fe–S-containing mitochondrial proteins. However, the role of IscU2 in tumor development remains unclear. Here, we demonstrated that IscU2 expression is much higher in human pancreatic ductal adenocarcinoma (PDAC) tissues than in adjacent normal pancreatic tissues. In PDAC cells, activated KRAS enhances the c-Myc-mediated *IscU2* transcription. The upregulated IscU2 stabilizes Fe–S cluster and regulates the activity of tricarboxylic acid (TCA) cycle enzymes α-ketoglutarate (α-KG) dehydrogenase and aconitase 2, which promote α-KG catabolism through oxidative and reductive TCA cycling, respectively. In addition to promoting mitochondrial functions, activated KRAS-induced and IscU2-dependent acceleration of α-KG catabolism results in reduced α-KG levels in the cytosol and nucleus, leading to an increase in DNA 5mC due to Tet methylcytosine dioxygenase 3 (TET3) inhibition and subsequent expression of genes including DNA polymerase alpha 1 catalytic subunit for PDAC cell proliferation and tumor growth in mice. These findings underscore a critical role of IscU2 in KRAS-promoted α-KG catabolism, 5mC-dependent gene expression, and PDAC growth and highlight the instrumental and integrated regulation of mitochondrial functions and gene expression by IscU2 in PDAC cells.

## Introduction

The Warburg effect refers to a phenomenon in which cancer cells prefer glycolysis for survival and proliferation even in the presence of sufficient oxygen^1,2^. Many studies have demonstrated that mitochondrial oxidative phosphorylation (OXPHOS) is also instrumental for cancer cell growth^3–5^. Tricarboxylic acid (TCA) cycle intermediates, such as citrate, malate, and oxaloacetate, are building blocks for lipid and nucleotide syntheses^6^. Other intermediates, including isocitrate, malate, and aspartate, are indispensable for the generation of nicotinamide adenine dinucleotide phosphate (NADPH) to counteract oxidative stress in cancer cells^7^. In addition, fumarate and succinate can promote cancer cell proliferation by stabilizing hypoxia-inducible factors (HIFs) and promoting DNA and histone methylation^8^.

Glucose and glutamine are the two major carbon sources that fuel the TCA cycle. Cancer cells exhibit elevated glucose and glutamine metabolism to promote cell proliferation. Under glucose-deficient conditions, glutamine can provide both carbon and nitrogen for macromolecular biosynthesis and glutathione for redox balance^9^. Oncogenes, such as MYC^10,11^ and KRAS^12^, can promote glutaminolysis by transcriptionally upregulating metabolic enzymes of glutaminolysis. In mitochondria, glutamine is converted to glutamate by glutaminase (GLS). Glutamate is then oxidatively deaminated to produce α-ketoglutarate (α-KG) by glutamate dehydrogenase (GLUD). Glutamate oxaloacetate transaminases 1 and 2 (GOT1/2) can also convert glutamate to α-KG, and this conversion is coupled with aspartate synthesis from oxaloacetate. α-KG then enters and fuels the TCA cycle. α-KG is also a key cofactor for dioxygenases, including Jumonji-domain-containing histone demethylases^13^. Previous reports showed that α-KG can inhibit breast cancer cell metastasis^14^ and promote tumor cell differentiation by inducing histone hypomethylation^15^ or attenuating Wnt signaling^16^. In addition, α-KG could antagonize the functions of fumarate and succinate and the oncometabolic activity of D-2-hydroxyglutarate (D-2-HG) in isocitrate dehydrogenase (IDH)-mutated cancer cells^17,18^. Thus, α-KG has multifaceted roles and acts as a key component of the TCA cycle and a cofactor for dioxygenases, which exhibit tumor-promoting and tumor-suppressing functions. However, how cancer cells coordinately inhibit the tumor-suppressing functions of α-KG and elevate the tumor-promoting function and mitochondrial metabolism of α-KG is largely unknown.

In this study, we showed that the iron–sulfur (Fe–S) cluster assembly protein IscU2 is transcriptionally upregulated by activated KRAS in PDAC cells in a mechanism dependent on KRAS-enhanced c-Myc expression. Upregulation of IscU2 expression increased α-KG catabolism and mitochondrial functions and reduced α-KG levels in the cytosol and nucleus of PDAC cells, leading to an increase in DNA 5mC and gene expression for tumor growth.

## Results

### Activated KRAS increases the catabolic rates of α-KG through TCA cycling in PDAC cells

**Fig. 1:**
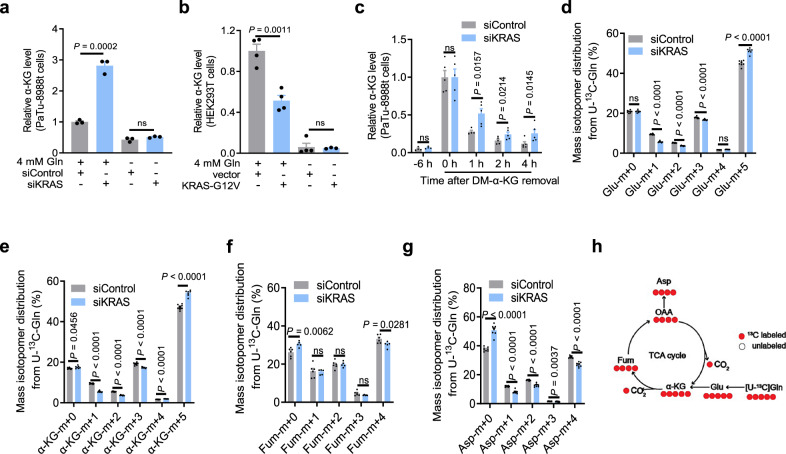
KRAS activation reduces α-KG level by enhancing TCA cycling in PDAC cells. **a** Relative α-KG levels in PaTu-8988t cells transfected with a control siRNA or KRAS siRNA and cultured in medium with or without Gln for 24 h. The determined α-KG levels were normalized to the numbers of viable cells (means ± SEM, *n* = 3). **b** Relative α-KG levels in HEK293T cells with or without expression of the human KRAS G12V mutant cultured in medium with or without 4 mM Gln for 24 h. The determined α-KG levels were normalized to the numbers of viable cells (means ± SEM, *n* = 4). **c** The indicated cells were cultured in medium without Gln for 24 h followed by pretreatment with DM-α-KG (7 mM) for 6 h. The α-KG levels were determined in the cells after DM-α-KG removal at 0, 1, 2, and 4 h (means ± SEM, *n* = 5). Data were normalized to the numbers of cells. See Supplementary Fig. S1j for a detailed experimental design. **d**–**g** U-^13^C-glutamine metabolic flux analyses in the PaTu-8988t cells. Mass isotopolog distributions of glutamate (**d**), α-KG (**e**), fumarate (**f**), and aspartate (**g**) are shown (means ± SEM, *n* ≥ 5). **h** Schematic model of U-13C-glutamine metabolism in the TCA cycle. Black arrows indicate oxidative metabolism. Red and white circles indicate labeled and unlabeled carbons, respectively. Statistical significance was determined by unpaired two-tailed Student’s *t*-test, ns not significant.

Oncogenic KRAS activates GOT1/2 to promote glutamine metabolism, which supports aspartate production in pancreatic ductal adenocarcinoma (PDAC) cells^12,19^. α-KG is one of the key intermediate metabolites during glutaminolysis; however, KRAS depletion led to the accumulation of α-KG in PDAC cells (Supplementary Fig. S1a), while overexpression of the KRAS G12V mutant significantly decreased the absolute content of α-KG in HEK293T cells (Supplementary Fig. S1b), indicating that KRAS plays a role in α-KG catabolism. To examine the regulation of α-KG levels in KRAS-mutated PDAC cells, we depleted KRAS in PaTu-8988t and PANC-1 PDAC cells and cultured cells in medium with or without glutamine (Supplementary Fig. S1c, d). We found that the α-KG levels were significantly increased in these cells (Fig. 1a; Supplementary Fig. S1e). In contrast, overexpression of the KRAS G12V mutant in HEK293T cells in the presence of glutamine (Supplementary Fig. S1f) significantly decreased the α-KG levels (Fig. 1b). Analyses of the α-KG levels in mice showed that the α-KG levels were reduced in PDAC tissues from the KRAS G12D/Trp53 null/Pdx1-cre (KPC) mice compared to those in normal pancreatic tissues (Supplementary Fig. S1g)^20^. Notably, the change in the α-KG level induced by KRAS depletion (Fig. 1a; Supplementary Fig. S1e) or KRAS G12V expression (Fig. 1b) was eliminated by glutamine deprivation in cultured medium. These results strongly suggest that KRAS activation promotes glutamine metabolism with reduced glutamine-derived α-KG levels in PDAC.

To determine whether the KRAS-reduced α-KG levels result from the inhibition of α-KG synthesis, we examined the mRNA levels of GLS, GOT1/2, and GLUD1/2, which are enzymes involved in the conversion of glutamine to α-KG (Supplementary Fig. S1h). Real-time PCR analyses showed that KRAS depletion reduced the expression of *GOT1* and *GLUD1* without altering *GLS* or *GOT2* expression (Supplementary Fig. S1i), suggesting that the KRAS-reduced α-KG levels is not due to decreased expression of the genes involved in α-KG production.

To determine whether KRAS reduces α-KG levels by promoting α-KG catabolism, we conducted glutamine deprivation experiments and added exogenous α-KG (Supplementary Fig. S1j), which constituted most of the intracellular α-KG (Fig. 1c). A time-course experiment showed that depletion of KRAS increased the α-KG levels in PaTu-8988t cells (Fig. 1c), suggesting that KRAS enhances α-KG catabolism. Consistent with this finding, ^13^C-glutamine metabolic tracing experiments showed that KRAS depletion significantly increased the levels of glutamate (m + 5) (Fig. 1d) and α-KG (m + 5) (Fig. 1e) and reduced the levels of glutamate (m + 1, + 2, + 3) and α-KG (m + 1, + 2, + 3) derived from the subsequent oxidative TCA cycle, further indicating that KRAS enhances α-KG catabolism instead of decreasing α-KG production from glutamate. In addition, KRAS depletion reduced the levels of fumarate (m + 4) and aspartate (m + 4) (Fig. 1f–h). These results strongly suggest that activated KRAS increases the catabolic rates of α-KG through TCA cycling in PDAC cells.

### Activated KRAS increases α-KG catabolism through c-Myc-upregulated IscU2 expression

To determine the mechanism underlying activated KRAS-increased α-KG catabolism, we examined TCA cycle enzymes (Supplementary Fig. S1h) and showed that KRAS depletion only moderately reduced the mRNA levels of the succinate dehydrogenase (SDH) genes *SDHA* and *SDHB* without altering other enzyme expression in PaTu-8988t cells (Supplementary Fig. S2a), suggesting that activated KRAS-increased α-KG catabolism is not induced by transcriptional alteration of TCA cycle enzyme expression.

**Fig. 2:**
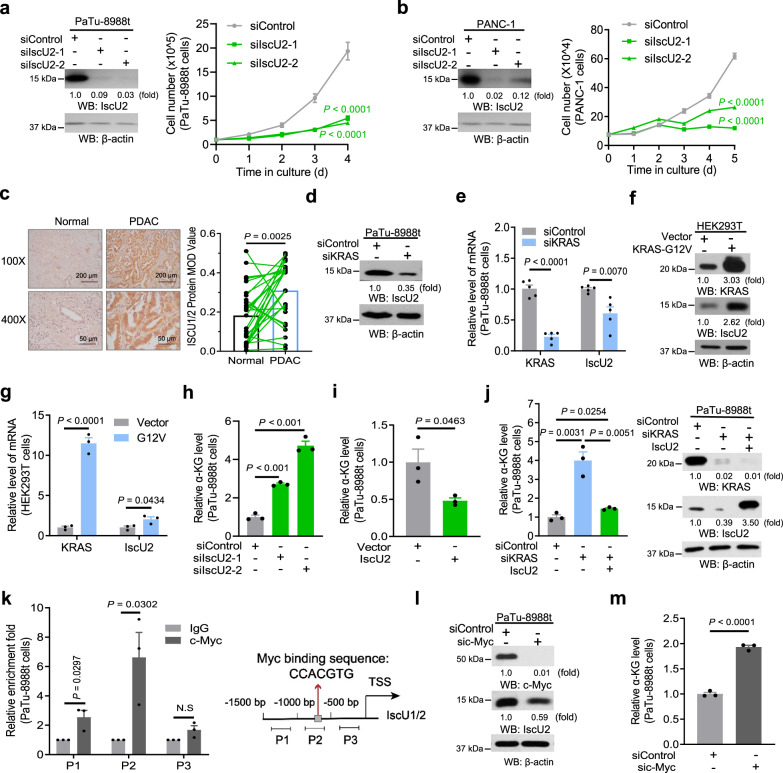
KRAS increases α-KG catabolism by promoting c-Myc-upregulated IscU2 expression in PDAC cells. **a**, **b** PaTu-8988t cells (**a**) and PANC-1 cells (**b**) were transfected with a control siRNA or IscU2 siRNA. Cell proliferation was examined (means ± SEM, *n* ≥ 3). Immunoblot analyses were performed with the indicated antibodies (left panel). **c** IHC analyses of 27 PDAC specimens and their paired adjacent normal pancreatic tissues were performed with an anti-IscU antibody. Representative images are shown (left panel). Comparative analysis of IscU expression between pancreatic tumor specimens and their adjacent normal tissues was performed (right panel). **d**, **e** Immunoblot analysis (**d**) for IscU2 and mRNA expression analysis (**e**) of *KRAS* and *IscU2* in PaTu-8988t cells transfected with a control siRNA or KRAS siRNA (means ± SEM, *n* = 5). **f** Immunoblot analysis of indicated proteins in HEK293T cells stably overexpressed empty control vector or the KRAS G12V mutant. **g** mRNA expression analysis of *KRAS* and *IscU2* in PaTu-8988t cells overexpressed with empty control vector or the KRAS G12V mutant (means ± SEM, *n* = 3). **h** Relative α-KG levels in PaTu-8988t cells transfected with a control siRNA or IscU2 siRNA (means ± SEM, *n* = 3). **i** Relative α-KG levels in PaTu-8988t cells expressed empty vector or IscU2 (means ± SEM, *n* = 3). **j** Relative α-KG levels in PaTu-8988t cells transfected with a control siRNA, KRAS siRNA, or KRAS siRNA with a vector expressing IscU2 (means ± SEM, *n* = 3). Immunoblot analyses were performed with the indicated antibodies. **k** ChIP analyses were performed with an anti-IgG or an anti-c-Myc antibody in PaTu-8988t cells. qPCR analyses were performed with three different pairs of primers targeted to the –1500 to –1000 bp (P1), –1000 to –500 bp (P2), and –500 to 0 bp (P3) regions of the IscU2 promoter (means ± SEM, *n* = 3). Gray box predicted binding site of c-Myc, TSS transcription start site, P primer. **l** PaTu-8988t cells were transfected with a control siRNA or c-Myc siRNA. Immunoblot analyses were performed with the indicated antibodies. **m** Relative α-KG levels in PaTu-8988t cells transfected with a control siRNA or c-Myc siRNA (means ± SEM, *n* = 3). Statistical significance was determined by unpaired two-tailed Student’s *t*-test, ns not significant.

Multiple mitochondrial proteins, including aconitase 2 (ACO2), NDUFS1/3 (a complex I component), SDHB, and UQCRFS1 (a complex III component), required Fe–S clusters for their functions^21^. Analyses of The Cancer Genome Atlas database (TCGA) and Genotype-Tissue Expression (GTEx) showed that PDAC tissues, compared to normal pancreatic tissues, exhibited highly increased mRNA expression of genes encoding Fe–S cluster assembly-related proteins, including *IscU* (which is the combined total mRNA level of the alternately spliced isoform cytosol-localized IscU1 and the mitochondria-localized IscU2), *IscU2*, *ISCA1*, *FDX1L*, *ISCA2*, *GLRX5*, *BOLA3*, and *NFU1* (Supplementary Fig. S2b). In normal pancreatic or PDAC tissues, the mRNA and protein levels of IscU1 were too low to be detected (Supplementary Fig. S2c, d), suggesting that IscU2 is the isoform primarily expressed in PDAC cells. To determine the roles of these Fe–S cluster assembly-related proteins in PDAC cell proliferation, we depleted all these proteins by expressing their siRNAs and showed that only depletion of IscU2 reduced the proliferation of PaTu-8988t and PANC-1 PDAC cells (Fig. 2a, b; Supplementary Fig. S2e–j)^22^, and this reduction was reversed by re-expression of IscU2 in cells (Supplementary Fig. S2k). We also determined cytosolic Fe–S cluster levels using a previously validated fluorescent detection system^23^ and results showed that depletion of IscU2 reduced Fe–S cluster levels more strongly as compared with depletion of IscA1, FDXIL, IscA2, GLRX5, BOLA3, and NFU1 (Supplementary Fig. S2l). IscU2 expression was much higher in the human PDAC tissues than in the adjacent normal pancreatic tissues, as detected by immunohistochemistry (IHC) (Fig. 2c).

The regulation of IscU2 expression by KRAS was further validated by the reduction and increase in the protein and mRNA levels of IscU2 induced by KRAS depletion (Fig. 2d, e) and overexpression of KRAS G12V (Fig. 2f, g), respectively. These results suggest that KRAS regulates *IscU2* transcription. Depletion of IscU2, acting similarly to KRAS depletion, increased the α-KG levels in PaTu-8988t and PANC-1 cells (Fig. 2h; Supplementary Fig. S3a), and this increase was eliminated by glutamine deprivation (Supplementary Fig. S3b). Consistently, NFS1 (a sulfur donor enzyme required for Fe–S assembly) depletion was also found able to suppress cell proliferation and increase α-KG levels in PaTu-8988t cells (Supplementary Fig. S3c–e), which highlighted that IscU2-mediated tumor-promoting effect relied on its Fe–S clusters assembly-associated function. In contrast, IscU2 overexpression decreased the α-KG levels (Fig. 2i). Notably, IscU2 overexpression abrogated the KRAS depletion-increased α-KG levels (Fig. 2j). These results strongly suggest that activated KRAS increases α-KG catabolism through upregulation of *IscU2* transcription.

Analyses of the *IscU2* promoter revealed that c-Myc is a potential transcriptional factor for *IscU2* transcription (Supplementary Table S1). Consistent with previous findings that c-Myc expression is regulated by KRAS^24^, KRAS depletion reduced c-Myc expression in PaTu-8988t cells (Supplementary Fig. S3f) whereas overexpression of KRAS G12V increased c-Myc expression in both HEK293T and BXPC3 PDAC cells (Supplementary Fig. S3g). Chromatin immunoprecipitation (ChIP) showed that c-Myc was predominantly localized at the –1000 to –500 bp region of the *IscU2* promoter (Fig. 2k). A luciferase reporter assay showed that the luciferase activity driven by the *IscU2* promoter was reduced by c-Myc depletion (Supplementary Fig. S3h) or enhanced by upregulation of c-Myc expression (Supplementary Fig. S3i). Notably, c-Myc depletion (Fig. 2l) in PaTu-8988t cells decreased the mRNA (Supplementary Fig. S3j) and protein levels of IscU2 (Fig. 2l) and induced the accumulation of α-KG (Fig. 2m). Correspondingly, increased α-KG level due to c-Myc depletion could be abrogated by overexpression of IscU2 (Supplementary Fig. S3k). Furthermore, downregulation of c-Myc level by a dual inhibition of ERK1/2 and ERK5 with SCH772984 and XMD8-92, could reverse KRAS-caused IscU2 upregulation (Supplementary Fig. S3l). While the glutamine concentration is limited in the tumor environment of PDAC^25^, which may minimize the generation of α-KG from glutamine, to ask if abnormal expression of KRAS, c-Myc, or IscU2 can also affect the level of α-KG, we then determined α-KG level in KRAS, c-Myc, or IscU2 depletion cells with limited glutamine supplementation (0.5 mM) (Supplementary Fig. S3m). Our results suggest that KRAS, c-Myc, and IscU2 axis could also be able to regulate α-KG level in physiological condition. Collectively, these results indicate that activated KRAS increases α-KG catabolism through c-Myc-upregulated IscU2 expression.

### Activated KRAS promotes α-KG catabolism through IscU2-enhanced DLST and ACO2 activity

**Fig. 3:**
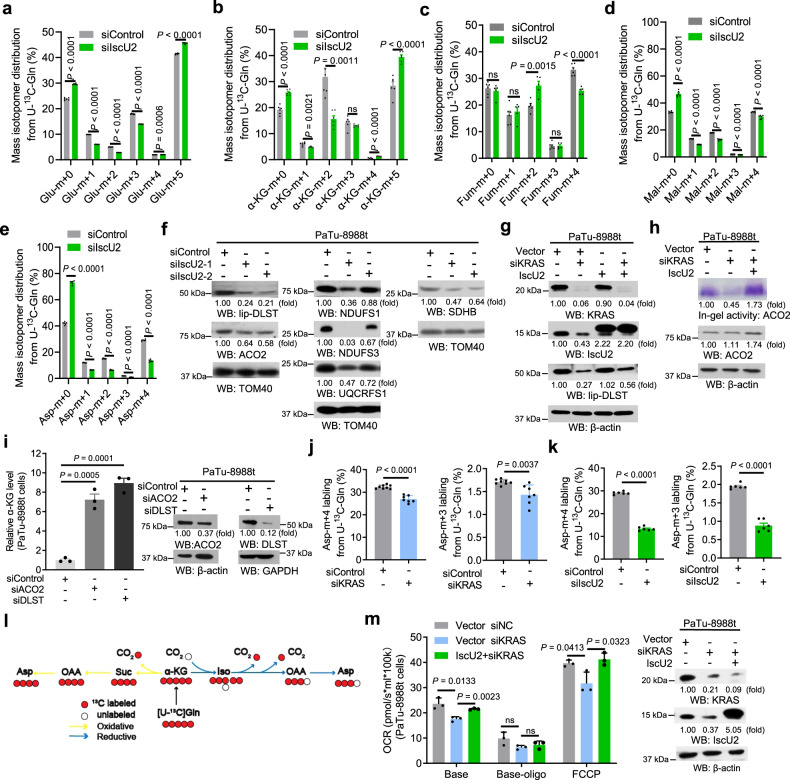
KRAS promotes the lipoylation of DLST and increases the activity of ACO2 in an IscU2 expression-dependent manner. **a**–**e** PaTu-8988t cells transfected with a control siRNA or IscU2 siRNA were cultured with 2 mM U-^13^C-glutamine for 24 h before metabolite extraction. Mass isotopomer distributions of glutamate (**a**), α-KG (**b**), fumarate (**c**), malate (**d**), and aspartate (**e**) are shown (means ± SEM, *n* ≥ 5). **f** PaTu-8988t cells were transfected with IscU2 siRNA. Immunoblot analyses were performed with the indicated antibodies. **g** PaTu-8988t cells transfected with a control siRNA, KRAS siRNA or KRAS siRNA with a vector expressing IscU2. Immunoblot analyses were performed with the indicated antibodies. **h** Mitochondrial ACO2 activity was analyzed by the in-gel activity assay. Immunoblot analyses were performed with the indicated antibodies. **i** PaTu-8988t cells were transfected with a control siRNA, ACO2 siRNA, or DLST siRNA. α-KG levels were determined and normalized to the numbers of cells (means ± SEM, *n* = 3). **j** PaTu-8988t cells transfected with a control siRNA or KRAS siRNA were cultured with 2 mM U-^13^C-glutamine for 24 h before metabolite extraction. Aspartate m + 3 and m + 4 labeling from U-^13^C-glutamine is shown (means ± SEM, *n* ≥ 7). **k** PaTu-8988t cells transfected with a control siRNA or IscU2 siRNA were cultured with 2 mM U-^13^C-glutamine for 24 h before metabolite extraction. Aspartate m + 3 and m + 4 labeling from U-^13^C-glutamine are shown (means ± SEM, *n* = 6). **l** Schematic model of oxidative and reductive TCA cycling of α-KG produced from U-^13^C-glutamine. Yellow and blue arrows indicate oxidative and reductive metabolism, respectively. Red circles indicate labeled carbons. **m** OCR of PaTu-8988t cells transfected with a control siRNA, KRAS siRNA or KRAS siRNA with a vector expressing IscU2 (means ± SEM, *n* = 3). Immunoblot analyses were performed with the indicated antibodies. Statistical significance was determined by unpaired two-tailed Student’s *t*-test, ns not significant.

To examine the effect of IscU2 expression on α-KG metabolism, we performed ^13^C-glutamine metabolic tracing experiments and showed that IscU2 depletion, similar to KRAS depletion, increased the levels of glutamate (m + 5) (Fig. 3a) and α-KG (m + 5) (Fig. 3b) accompanied by reduced levels of glutamate (m + 1, + 2, + 3) (Fig. 3a), α-KG (m + 1, + 2) (Fig. 3b), and fumarate (m + 4), malate (m + 4) and aspartate (m + 4) (Fig. 3c–e) in PaTu-8988t cells. These results strongly suggest that upregulated IscU2 expression by activated KRAS increases catabolism of α-KG in PDAC cells.

IscU2 is a scaffold protein critical for assembly of Fe–S clusters and the functions of multiple Fe–S-containing mitochondrial proteins^21^. IscU2 depletion reduced the expression of all these Fe–S-dependent mitochondrial proteins, including lipoylated DLST (lip-DLST), ACO2, NDUFS1/3 (a complex I component), SDHB (a complex II component), and UQCRFS1 (a complex III component), in PaTu-8988t (Fig. 3f) and PANC-1 (Supplementary Fig. S4a) cells. Notably, activated DLST itself does not contain Fe–S clusters but requires lipoic acid for lipoylation, and synthesis of lipoic acid requires Fe–S clusters-containing lipoic acid synthase^26^. Treatment with the proteasome inhibitor MG132 partially restored DLST expression (Supplementary Fig. S4b), suggesting that mitochondrial protein DLST without lipoylation is unstable and subject to degradation. Similar to IscU2 depletion, KRAS depletion, which did not affect the mRNA levels of *DLST* (Supplementary Fig. S2a), reduced the protein levels of IscU2 and lip-DLST (Fig. 3g). Notably, KRAS depletion-induced reduction of DLST was resecured by overexpression of IscU2. Moreover, IscU2 overexpression was found able to increase ACO2 protein level and activity in PaTu-8988t cells with KRAS depletion (Fig. 3h), which probably due to overload of Fe–S cluster supplementation^27,28^. We identified that at least 75% reduction of IscU2 expression in PDAC cells was required to significantly reduce ACO2 protein level (Supplementary Fig. S4c, d). In contrast, IscU2 overexpression, which likely increased Fe–S cluster level in a large scale thereby reducing Fe–S clusters-unbounded ACO2 amount, increased ACO2 level in PaTu-8988t cells (Supplementary Fig. S4e). KRAS depletion generally led to ~60% decrease of IscU2 (Figs. 2d, 3g, m), which was not enough to reduce the protein level of ACO2, but could reduce ACO2 activity (Fig. 3h), suggesting that ACO2 stability is regulated by Fe–S cluster levels in an IscU2 protein expression-dependent manner and is decreased only under certain Fe–S clusters threshold. To assess the impact of KRAS on Fe–S cluster formation, we measured Fe–S cluster levels in PANC-1 cells with or without KRAS depletion and HEK293T cells expressed empty vector or KRAS mutant using a validated Fe–S cluster formation assay^23^. The results showed that the Fe–S cluster level was decreased in PANC-1 cells with KRAS depletion (Supplementary Fig. S4f), but increased in HEK293T cells expressing the KRAS G12V mutant (Supplementary Fig. S4g). These results strongly suggest that KRAS promotes the lipoylation of DLST and increases the activity of ACO2 in an IscU2 expression-dependent manner.

Of note, inhibition of complex I with rotenone (ROT) (Supplementary Fig. S4h) and complex III with antimycin A (AA) (Supplementary Fig. S4i) did not recapitulate the effect of IscU2 depletion on α-KG accumulation. In contrast, inhibition of ACO2 with fluorocitrate (Supplementary Fig. S4j) or DLST with devimistat (CPI-613) (Supplementary Fig. S4k) or depletion of these enzymes substantially increased the α-KG levels (Fig. 3i). In line with the role of DLST and ACO2 as key enzymes for oxidative and reductive TCA cycling of α-KG (Supplementary Fig. S1h), respectively, ^13^C-glutamine metabolic tracing experiments showed that depletion of KRAS (Fig. 3j) or IscU2 (Fig. 3k) resulted in inhibition of oxidative and reductive TCA cycling of glutamine reflected by reduction of m + 4- and m + 3-labeled amounts of aspartate and oxaloacetic acid (OAA), respectively (Fig. 3l). These results strongly suggested that activated KRAS promotes α-KG catabolism through IscU2-enhanced DLST and ACO2 activities. Analyses of mitochondrial functions revealed that KRAS depletion reduced the oxygen consumption rate (OCR), and this reduction was largely resecured by overexpression of IscU2 (Fig. 3m), indicating that KRAS-mediated IscU2 upregulation promotes α-KG catabolism and TCA cycling.

To confirm whether KRAS and IscU2 regulate citrate through ACO2, we determined the citrate level in PaTu-8988t cells with or without depletion of IscU2 or KRAS, and showed that IscU2 or KRAS depletion led to the decrease of citrate level (Supplementary Fig. S4l). In addition, we showed that citrate level was increased in PaTu-8988t cells with ACO2 depletion (Supplementary Fig. S4m) and decreased in HEK293T cells with overexpression of ACO2 (Supplementary Fig. S4n). As expected, ACO2 depletion in PaTu-8988t cells with depletion of IscU2 or KRAS increased citrate level (Supplementary Fig. S4o, p), suggesting that ACO2 plays a role in regulation of citrate production in tumor cells expressing activated KRAS.

### Activated KRAS reduces the α-KG levels in the cytosol and nucleus of PDAC cells through IscU2-enhanced mitochondrial catabolism of α-KG

**Fig. 4:**
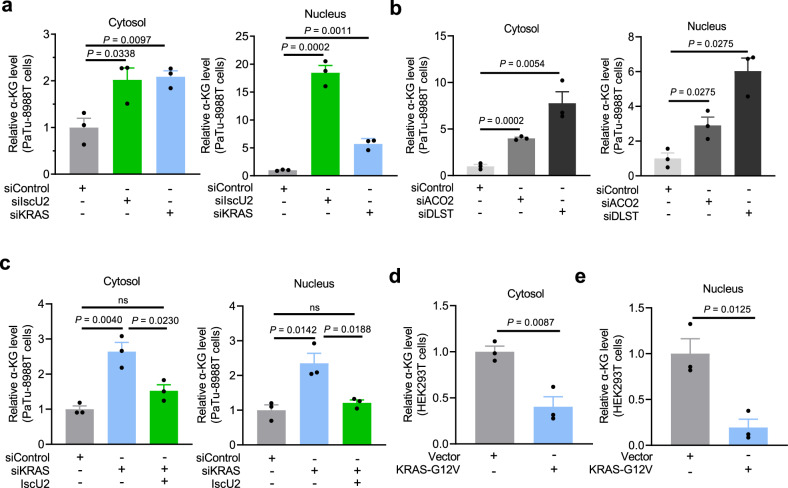
KRAS activation reduces α-KG levels in the cytosol and nucleus of PDAC cells. **a** PaTu-8988t cells were transfected with a control siRNA, IscU2 siRNA, or KRAS siRNA. Cytosolic and nuclear α-KG levels were determined and normalized to the numbers of cells (means ± SEM, *n* = 3). **b**, **c** PaTu-8988t cells were transfected with a control siRNA, ACO2 siRNA, or DLST siRNA (**b**). PaTu-8988t cells were transfected with a control siRNA, KRAS siRNA, or KRAS siRNA with a vector expressing IscU2 (**c**). Both cytosolic and nuclear α-KG levels were determined and normalized to the number of cells (means ± SEM, *n* = 3). **d**, **e** HEK293T cells stably overexpressed KRAS G12V. Cytosolic (**d**) and nuclear (**e**) α-KG levels were determined and normalized to the numbers of cells (means ± SEM, *n* = 3). Statistical significance was determined by unpaired two-tailed Student’s *t*-test, ns not significant.

The malate-aspartate shuttle simultaneously imports malate from the cytosol to the mitochondrial matrix and exports α-KG from the mitochondrial matrix to the cytosol (Supplementary Fig. S5)^29^. We next examined whether activated KRAS-induced and IscU2-promoted α-KG catabolism regulate the α-KG levels in the cytosol and nucleus of PDAC cells. We found that depletion of KRAS or IscU2 strongly increased the α-KG levels in both the cytosol and the nucleus of PaTu-8988t cells (Fig. 4a). Similar results were also obtained by depletion of DLST and ACO2 (Fig. 4b). Notably, KRAS depletion increased the α-KG levels in the cytosol and the nucleus, and this increase was inhibited by overexpression of IscU2 (Fig. 4c). In contrast, the expression of KRAS G12V reduced the α-KG levels in the cytosol (Fig. 4d) and nucleus (Fig. 4e) of PDAC cells. These results suggest that activated KRAS reduces α-KG levels in the cytosol and nucleus of PDAC cells through IscU2-enhanced mitochondrial catabolism of α-KG.

### KRAS promotes DNA 5mC-regulated gene expression in PDAC cells through IscU2-enhanced α-KG catabolism

**Fig. 5:**
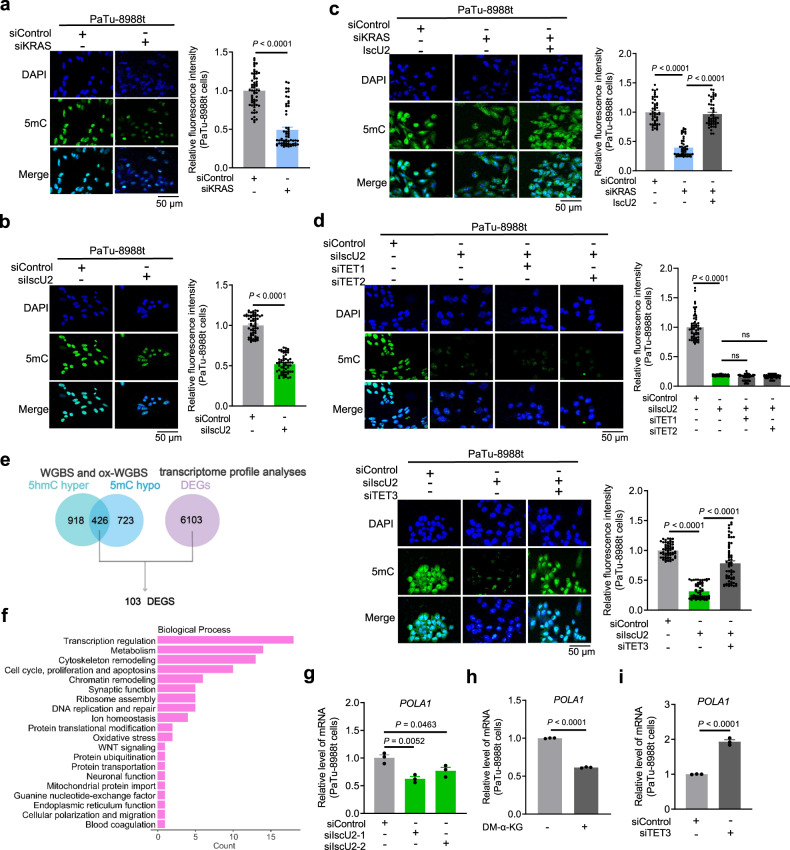
KRAS promotes DNA 5mC in PDAC cells through IscU2-enhanced α-KG catabolism. **a**–**d** PaTu-8988t cells were transfected with a control siRNA, KRAS siRNA (**a**), IscU2 siRNA (**b**), KRAS siRNA with a vector expressing IscU2 (**c**), or vectors expressing IscU2 and TET3 siRNAs (**d**). Immunofluorescence analyses were performed with the indicated antibodies. Nuclei were stained with DAPI. Quantification performed from 3 experiments with 60 cells quantified for each condition. Scale bars represent a distance of 50 µm. **e** Workflow of oxidative bisulfite sequencing with transcriptome profile analyses; 5hmC hyper/5mC hypo: higher 5hmC and lower 5mC in IscU2-depleted cells when compared with the control PaTu-8988t cells. DEGs: differentially expressed genes between the PaTu-8988t cells transfected with IscU2 siRNA and the control PaTu-8988t cells. **f** A bar plot showing the functional enrichment analysis for DEGs between the PaTu-8988t cells transfected with IscU2 siRNA and the control PaTu-8988t cells. **g**–**i** qPCR analysis of *POLA1* in the PaTu-8988t cells transfected with IscU2 siRNA (**g**), the PaTu-8988t cells treated with or without 7 mM DM-α-KG (**h**), or the PaTu-8988t cells transfected with TET3 siRNA (**i**) (means ± SEM, *n* = 3). Statistical significance was determined by unpaired two-tailed Student’s *t*-test, ns not significant.

In addition to its critical role in mitochondria, α-KG is a key cofactor for dioxygenases, such as TET methylcytosine dioxygenase family members (TET1–3) that convert DNA 5-methylcytosine (5mC) to 5-hydroxymethylated cytosine (5hmC), thereby inhibiting DNA 5-methylcytosine^13^. In contrast, succinate and fumarate are two catabolic products of α-KG, which suppress dioxygenases activity by acting as competitive inhibitors of α-KG^30^. Inhibiting α-KG catabolism due to IscU2, c-Myc, or KRAS depletion not only induced α-KG but also led to the increased ratios of α-KG/succinate and α-KG/fumarate (Supplementary Fig. S6a–c), indicating a regulatory role of IscU2, c-Myc, or KRAS on DNA 5mC. This hypothesis was evidenced while depletion of KRAS (Fig. 5a; Supplementary Fig. S6d) or IscU2 (Fig. 5b; Supplementary Fig. S6e) reduced genomic 5mC levels in PaTu-8988t and PANC-1 cells, which recapitulated the effect induced by treatment of these cells with exogenous α-KG or depletion of c-Myc (Supplementary Fig. S6f–h). In contrast, increased genomic 5hmC was found in cells with depletion of KRAS, IscU2, or c-Myc (Supplementary Fig. S6i). Moreover, to exclude other factors which may contribute to the change of 5mC level, we determined the mRNA levels of DNMTs (DNMT1, DNMT3A and DNMT3B) and the ratio of S-adenosylhomocysteine (SAH)/S-adenosylmethionine (SAM), and showed that both the expression of DNMTs and the SAH/SAM ratio were not changed in IscU2 depleted cells (Supplementary Fig. S6j, k). These results further suggest that KRAS, c-Myc, or IscU2 regulates the availability of α-KG to finetune the DNA methylation.

Notably, the KRAS depletion-mediated reduction in genomic DNA 5mC levels was reversed by IscU2 overexpression (Fig. 5c) or depletion of TET3, but not TET1 or TET2 (Supplementary Fig. S6l). Likewise, depletion of TET3, but not TET1 or TET2, reduced the inhibitory effect of IscU2 depletion on DNA 5mC (Fig. 5d). The favored preference of TET3 in the regulation of DNA methylation is probably due to the highest TET3 level among all TETs in PDAC cells (Supplementary Fig. S6m), and TET3 rather than TET1 and TET2 plays a major role in the alteration of 5mC (Supplementary Fig. S6n, o). These results indicate that activated KRAS promotes DNA 5mC in PDAC cells through IscU2-enhanced α-KG catabolism and subsequent TET3 inhibition.

To investigate the effect of the IscU2-mediated increase in DNA 5mC on gene expression, we performed whole-genome bisulfite sequencing (WGBS), oxidative whole-genome bisulfite sequencing (ox-WGBS) (Supplementary Dataset S1), and RNA sequencing (Supplementary Dataset S2) to evaluate genome-wide differentially methylated cytosines (DmCs), differentially hydroxymethylated cytosines (DhmCs), and gene transcription in PDAC cells. A total of 426 genes were found to have lower 5mC modification but higher 5hmC modification in the IscU2-depleted cells than in the control cells (Fig. 5e; Supplementary Dataset S3), of which 103 were differentially expressed genes (DEGs) regulated by IscU2 depletion (Supplementary Dataset S4). Among 103 genes, 93 genes are involved in the regulation of critical cellular activities, including transcription, metabolism, cell cycle and survival regulation, chromatin remodeling, and DNA replication and repair (Fig. 5f; Supplementary Dataset S4). Notably, one of these genes is DNA polymerase alpha 1 (*POLA1*, also known as the alpha DNA polymerase-primase complex), which plays an essential role in the initiation of DNA synthesis and cell cycle progression^31^, and was hypo-methylated at gene body in IscU2-depleted cells when compared with control cells (Supplementary Dataset S1). Real-time PCR analysis showed that IscU2 depletion (Fig. 5g; Supplementary Fig. S6p) or exogenous α-KG treatment (Fig. 5h) reduced the *POLA1* mRNA levels in PaTu-8988t or PANC-1 cells, whereas TET3 depletion increased *POLA1* expression (Fig. 5i). These results indicate that activated KRAS promotes DNA 5mC-regulated expression of genes, including *POLA1*, in PDAC cells through IscU2-enhanced α-KG catabolism.

### IscU2 expression upregulated by activated KRAS promotes α-KG catabolism and subsequent DNA 5mC-dependent PDAC cell proliferation and tumor growth in mice

**Fig. 6:**
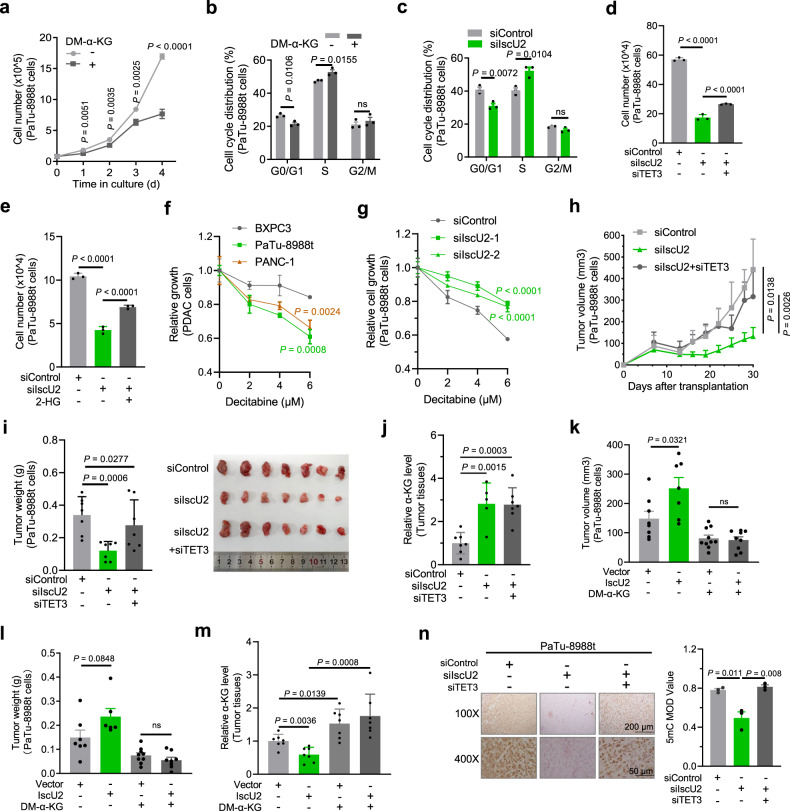
IscU2 promotes α-KG catabolism and subsequent DNA 5mC-dependent PDAC cell proliferation and tumor growth in mice. **a** Cell proliferation of the PaTu-8988t cells with or without DM-α-KG (7 mM) treatment (means ± SEM, *n* = 3). **b** Determination of the cell cycle stage of the PaTu-8988t cells treated with or without DM-α-KG (7 mM) for 48 h (means ± SEM, *n* = 3). **c** The PaTu-8988t cells transfected with a control siRNA or IscU2 siRNA were plated for 48 h before determination of the cell cycle stage (means ± SEM, *n* = 3). **d**, **e** The PaTu-8988t cells were transfected with a control siRNA, IscU2 siRNA, and TET3 siRNA (**d**), or IscU2 siRNA in combination with 100 μM octyl-(R)-2HG (**e**). The cells were plated for 4 days before measurement of cell proliferation (means ± SEM, *n* = 3). **f** BXPC3, PaTu-8988t, and PANC-1 cells were seeded in 12-well plates at a density of 2 × 10^4^ cells/well, respectively. After 48 h of culture, cells were treated with different concentrations of decitabine and cultured for another 48 h. A relative value was obtained by comparing decitabine-treated cells with decitabine-free cells at final time point. (means ± SEM, *n* = 3). **g** PaTu-8988t cells transfected with a control siRNA or IscU2 siRNA were seeded in 12-well plates at a density of 2 × 10^4^ cells/well. After 48 h of culture, the cells were treated with different concentrations of decitabine and cultured for another 48 h. A relative value was obtained by comparing decitabine-treated cells with decitabine-free cells at final time point. (means ± SEM, *n* = 3). **h**–**j** The PaTu-8988t cells (5 × 10^6^) transfected with control siRNA, IscU2 siRNA, and TET3 siRNA were subcutaneously injected into the flank regions of athymic nude mice. When the tumors (1 week after subcutaneous injection) were established, the volume of the tumors were measured every two days (**h**). And the tumor weights (**i**) and α-KG levels (**j**) were measured after 4 weeks of tumor growth (means ± SEM, *n* ≥ 5). **k**–**m** PaTu-8988t cells (5 × 10^6^) with or without overexpression of IscU2 were injected into the flank regions of athymic nude mice. When the tumors were established, these mice were randomly divided into two groups and intraperitoneally injected with dimethyl α-KG (DM-α-KG) (600 mg/kg) or PBS daily. The mice were examined for 4 weeks of tumor growth after injection. The tumor volumes were calculated (**k**). The tumor weights (**l**) and the α-KG levels (**m**) were measured. (means ± SEM, *n* ≥ 6). **n** Immunohistochemical analyses of tumor samples were performed with an anti-DNA 5mC antibody. Representative images are shown (left panel). The 5mC levels in the tumor samples were determined (means ± SEM, *n* = 3). Statistical significance was determined by unpaired two-tailed Student’s *t*-test, ns not significant.

To determine the role of α-KG catabolism in PDAC cell proliferation, we treated PDAC cells with exogenous α-KG and showed that α-KG inhibited cell proliferation (Fig. 6a; Supplementary Fig. S7a), colony formation (Supplementary Fig. S7b) and sphere formation (Supplementary Fig. S7c) without obviously alteration of apoptosis (Supplementary Fig. S7d, e). Consistent with the exogenous α-KG-reduced POLA1 expression (Fig. 5h), exogenous α-KG treatment increased the number of PDAC cells arrested in S phase (Fig. 6b; Supplementary Fig. S7f). Similarly, IscU2 depletion reduced PaTu-8988t and PANC-1 cell proliferation (Fig. 2a, b) and arrested the cells at S-phase (Fig. 6c; Supplementary Fig. S7g) without affecting cell apoptosis (Supplementary Fig. S7h). In addition, IscU2 depletion- or KRAS depletion-inhibited proliferation or colony formation of PDAC cells was partially alleviated by TET3 depletion (Fig. 6d; Supplementary Fig. S7i–k). The partial rescue by TET3 depletion suggested that the regulation of other α-KG-dependent proteins is also involved in cell proliferation. Notably, the addition of octyl-(R)-2-HG, a competitive metabolic inhibitor of α-KG, substantially restored the proliferation of the IscU2-depleted PDAC cells (Fig. 6e), confirming that IscU2 regulates cell proliferation and cell cycle progression in an α-KG-dependent manner. To differentiate the effects of mitochondrial dysfunction and α-KG dysregulation induced by IscU2 on cell proliferation, we cultured PaTu-8988t cells under hypoxic conditions to exclude the effect from dysregulation of electron transport chain. We showed that IscU2 depletion was still able to inhibit the cell proliferation (Supplementary Fig. S7l), highlighting a role of IscU2-regulated α-KG levels in PDAC cell proliferation. Moreover, we found that BXPC3 cells with low IscU2 expression were less sensitive to decitabine (a clinical approved DNA methyltransferase inhibitor) treatment than PaTu-8988t and PANC1 cells with high IscU2 expression (Fig. 6f; Supplementary Fig. S7m). In addition, IscU2 depletion rendered PaTu-8988t cells resistant to decitabine treatment compared to the parental cells (Fig. 6g), indicating that IscU2 expression level may be a potential molecular biomarker for treating PDAC with DNMT inhibitors. Together, these results indicate that IscU2 expression upregulated by activated KRAS promotes α-KG catabolism and subsequent DNA 5mC-dependent PDAC cell proliferation and cell cycle progression.

We next examined the role of IscU2 in PDAC growth in mice by subcutaneous injection of PaTu-8988t or PANC-1 cells with or without IscU2 depletion or overexpression. IscU2 depletion strongly reduced tumor growth (Fig. 6h, i; Supplementary Fig. S7n–p), whereas IscU2 overexpression enhanced tumor development (Fig. 6k, l). IscU2 depletion increased α-KG level and reduced tumor growth, and the IscU2 depletion-reduced tumor growth was largely alleviated by TET3 depletion (Fig. 6h–j; Supplementary Fig. S7q, r). Notably, intraperitoneal injection of α-KG inhibited PaTu-8988t-derived tumor growth and eliminated the growth acceleration resulting from IscU2 overexpression (Fig. 6k–m). IHC analyses of DNA 5mC in tumor tissues showed that IscU2 depletion in the mice decreased the level of DNA 5mC, and this decrease was alleviated by TET3 depletion (Fig. 6n). These results strongly suggest that IscU2 expression is required for activated KRAS-promoted α-KG catabolism, DNA 5mC, and tumor growth in mice.

## Discussion

**Fig. 7:**
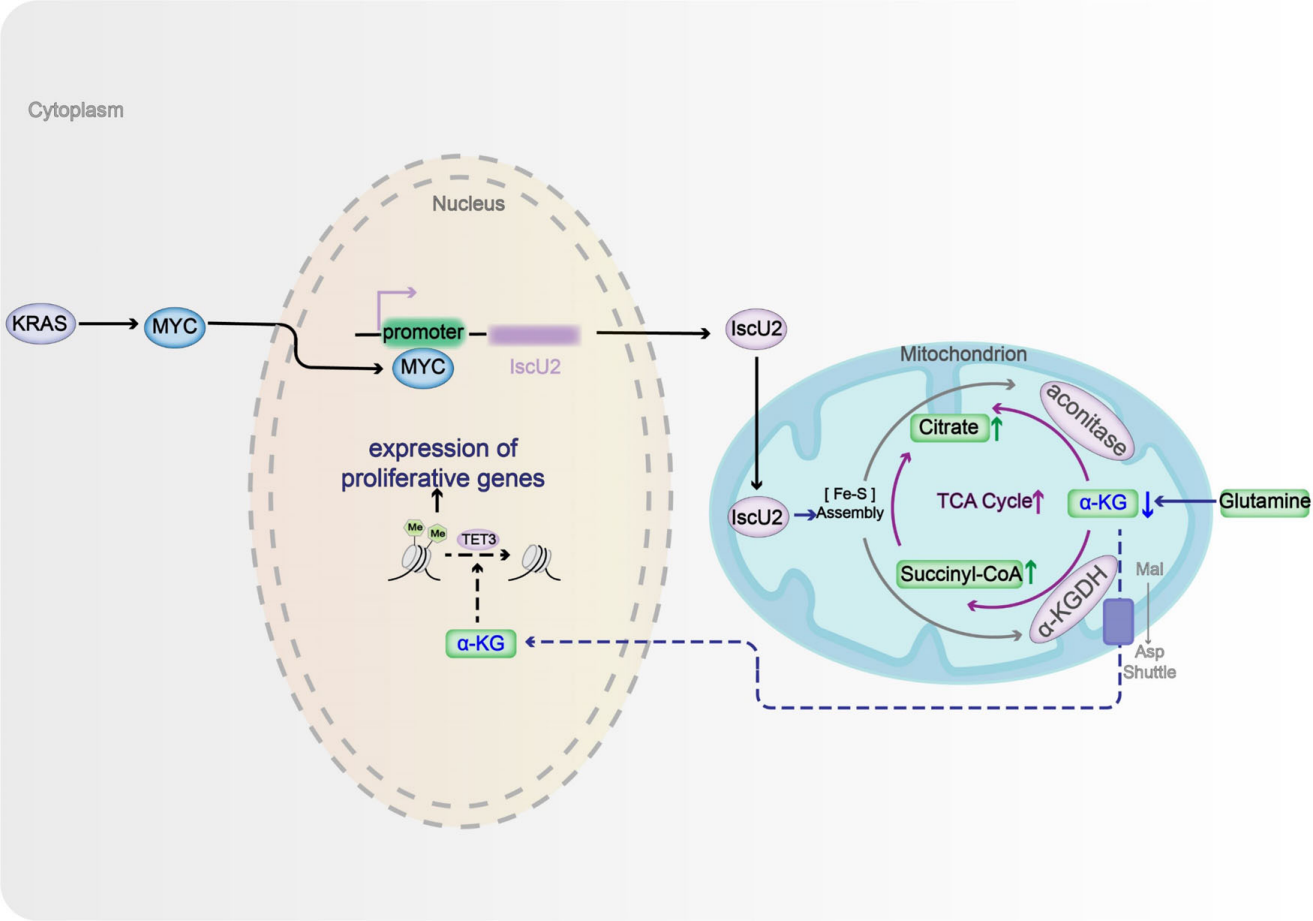
KRAS/c-Myc promotes DNA 5mC through IscU2-enhanced α-KG catabolism. KRAS-induced and c-Myc-mediated IscU2 upregulation stabilizes the expression of the Fe-S cluster-dependent TCA enzymes DLST and ACO2, thereby increasing α-ketoglutarate catabolism. This leads to the reduction of α-KG in the cytosol and nucleus of PDAC cells, which results in an increase in DNA 5mC due to TET3 inhibition.

KRAS activation is pivotal for the conversion of glutamine to α-KG by increasing the function of GOT1/2 or glutamate dehydrogenase 1, thereby promoting glutaminolysis and PDAC development^22^. However, α-KG was reported to have both tumor-promoting and tumor-inhibiting functions, and the mechanisms have not been fully elucidated^15,16,32–34^. Here, we showed that KRAS activation promotes α-KG catabolism through enhanced TCA cycling. Mechanistically, activated KRAS transcriptionally upregulated IscU2 expression through KRAS-enhanced expression of c-Myc, which specifically bound the promoter region of *IscU2*. Increased IscU2 expression stabilized the expression of the Fe–S cluster-dependent TCA enzymes DLST and ACO2, at least partially through inhibition of proteasome-mediated protein degradation. Enhanced expression of DLST and ACO2 increased the catalysis of α-KG in oxidative and reductive TCA cycling, respectively, thereby promoting α-KG catabolism and mitochondrial functions. In addition, activated KRAS-induced and IscU2-dependent acceleration of α-KG catabolism resulted in reduced α-KG levels in the cytosol and nucleus of PDAC cells and subsequent inhibition of TET3-dependent DNA 5hmC. The increased DNA 5mC level and expression of genes including *POLA1* promoted PDAC cell proliferation, colony formation, spheroid growth, and tumor development in mice (Fig. 7).

IscU2 is a scaffold protein critical for the assembly of Fe–S clusters and the functions of Fe–S-containing mitochondrial proteins, such as ACO2, α-KGDH, NDUFS1/3, SDHB, and UQCRFS1^21^. We showed that IscU2 regulates the stabilities of these Fe–S-containing proteins, indicating the regulatory role of Fe–S biosynthesis in the enhanced mitochondrial functions in tumor cells; however, IscU2 suppression was found to be able to decrease the activity of mitochondrial complex I and force an adaption to hypoxia in breast cancer cells^22,35^. Moreover, *ISCU* mutations were found to induce mitochondrial myopathy due to impairment of mitochondrial respiration^36^. Although other physiological consequences due to IscU2 abnormality is less studied, how IscU2 regulates mitochondrial function via Fe–S-containing mitochondrial proteins is very well established. One of the clear mechanisms is that IscU2 forms a Fe–S transfer complex with HSC20 and HSPA9 for Fe–S transfer^37^.

Given that IscU2 regulates not only α-KG catabolism but also many mitochondrial function-dependent cellular activities^38^, the partial rescue of IscU2 depletion-inhibited PDAC cell proliferation by TET3 depletion suggested multiple roles of IscU2, including suppression of α-KG-promoted DNA 5hmC in tumor promotion, and promotion of mitochondrial electron transfer coupled cancer cell proliferation^39^. Thus, our findings uncovered a previously unknown connection between Fe–S metabolism and PDAC tumor growth and underscored the critical and integrated regulation of mitochondrial functions and gene expression by IscU2. While KRAS active mutations are required for the development of many cancer types including non-small-cell lung carcinoma, colorectal cancer, and PDAC^40^. It is very much predictable that such mechanism was shared by these cancer types. Further, a previously described dual inhibition of MAPK signaling with chemicals of SCH772984 and XMD8-92^24^, which induced a dramatic degradation of c-Myc in KRAS activated cancer cells, can be a qualified strategy for the treatment of PDAC with KRAS activation.

Collectively, given that IscU2 is overexpressed in PDAC tissues compared to their adjacent normal tissues, our findings provide a novel potential approach for PDAC treatment by intervening in IscU2-mediated cellular activities.

## Materials and Methods

### Materials

L-glutamine, dimethyl alpha-ketoglutaric acid, U-^13^C-glutamine, octyl-(R)-2HG, rotenone, oligomycin, and 2-[2-[4-(trifluoromethoxy)phenyl] hydrazinylidene]-propanedinitrile were purchased from Sigma-Aldrich (MO, USA). Fluorocitrate was prepared from DL-fluorocitric acid barium salt (Sigma-Aldrich) as previously described^41^. Antimycin A was purchased from Abcam (MA, USA). Devimistat (CPI-613) was purchased from Selleck (TX, USA). MG132 was purchased from MCE (NY, USA). Antibodies recognizing β-actin (sc-47778; 1:5000), GAPDH (sc-32233; 1:1000), and NDUFS3 (sc-374282; 1:1000) were purchased from Santa Cruz Biotechnology (TX, USA). Antibodies recognizing IscU (#14812; 1:1000), NDUFSC1 (#12444-1-AP; 1:1000), and TOM40 (#18409-1-AP; 1:1000) were purchased from Proteintech (IL, USA). Antibodies recognizing UQCRFS1 (ab191078; 1:1000), aconitase 2 (ab110321; 1:1000), SDHB (ab14714; 1:1000), and α-KGDH (ab58724; 1:1000) were purchased from Abcam (Cambridge, United Kingdom). The secondary anti-mouse (#7076; 1:2000) or anti-rabbit (#7074; 1:2000) IgG antibodies and an anti-Myc antibody (#2276; 1:1000) were purchased from Cell Signaling Technology (MA, USA).

### Human tumor samples

Human PDAC specimens, including twenty-seven paired formalin-fixed, paraffin-embedded sections of primary PDAC specimens and adjacent normal pancreatic tissues (5 μm thick), were obtained from the First Affiliated Hospital of Wenzhou Medical University. This study was approved by the Ethics Committee of Wenzhou Medical University (2017).

### Animals

Nude mice (male, 4 weeks) were purchased from Beijing Vital River Lab Animal Technology Co., Ltd. (Beijing, China) and housed in groups in a specific-pathogen-free facility in the Laboratory Animal Center of Wenzhou Medical University. All animal studies were approved by the Animal Care Committee of Wenzhou Medical University and performed according to the protocol of the Laboratory Animal Center of Wenzhou Medical University.

### Cell culture

The PDAC cell lines PaTu-8988t, PANC-1, and BXPC3 were purchased from the Cell Resource Center (Chinese Academy of Sciences, Shanghai, China). Short tandem repeat loci analysis was used to authenticate the cell lines (Genetic Testing Biotechnology Corporation, Jiangsu, China). HEK293T cells were a gift from Dr. Haihua Gu (Wenzhou Medical University, Wenzhou, China). Cells were maintained in high-glucose Dulbecco’s modified Eagle’s medium (DMEM) with 12% calf serum (Sigma-Aldrich) for PaTu-8988t, PANC-1, and HEK293T cells or 10% fetal bovine serum (Clark Bioscience, DE, USA) for BXPC3 cells, with antibiotics (100 U/mL penicillin and 0.1 mg/mL streptomycin, Beyotime, Shanghai, China) and 2.5 ng/mL amphotericin B (Sangon Biotech, Shanghai, China) at 37 °C in an atmosphere of 5% CO_2_ (Thermo Fisher Scientific, MA, USA). Mycoplasma contamination was excluded using the MycoAlert PLUS Mycoplasma Detection Kit (Lonza Bioscience, Basel, Switzerland).

### Transfection and infection

siRNA transfection and transfection of plasmids were performed using Lipofectamine™ RNAiMAX (Thermo Fisher Scientific) and Lipofectamine™ 3000 (Thermo Fisher Scientific), respectively, according to the manufacturer’s instructions. Lentivirus particles for infection were produced by cotransfecting HEK293T cells with one of the expression plasmids and two packaging plasmids (pMD2G and pSPAX2) at a ratio of 1:1:2^42^. Infectious lentivirus particle-containing medium was collected at 72 h after transfection and centrifuged and filtered through a 0.45 μm filter (Millipore, Burlington, MA, USA). HEK293T or PDAC cells infected with lentiviral particles were screened with 2 μg/mL puromycin (Sangon Biotech) for 10 days prior to the following experiments.

### DNA construction and mutagenesis

Full-length open reading frames of mutant human KRAS (G12V, active form of KRAS, NM_033360.4), IscU1 (NM_014301.4) and IscU2 (NM_213595.4) with a myc-tag at the C-terminus and c-Myc (NM_002467.6) were synthesized by Tsingke Biological Technology (Hangzhou, China) and cloned into the lentiviral pLVX-Puro backbone (Cyagen, Guangzhou, Guangdon, China). The shRNAs used in this study were synthesized by Tsingke Biological Technology according to the following sequences: control shRNA, GGTAGCGACTAAACACATCAA; *IscU2* shRNA, GAAAAGGTTGTTGATCATTATCTC; and *KRAS* shRNA, TGGTCCTAGTAGGAAATAA. siRNA sequences and manufacturers details of *KRAS, c-Myc, IscU1/2, ISCA1, FDX1L, ISCA2, GLRX5, BOLA3, NFU1, TET1, TET2, TET3, DLST, and ACO2* are listed in Supplementary Table S2.

### α-KG, succinate, fumarate, and citrate measurement

α-KG, succinate, fumarate, and citrate were determined using α-KG Assay Kit (Abcam), Succinate Assay Kit (Abcam), Succinate Assay Kit (Abcam), and citrate Assay Kit (Abcam), respectively. Briefly, cells were rinsed with phosphate-buffered saline (PBS) for 3 times, and 5 × 10^6^ cells were harvested and resuspended in a mixture of 200 μL of cold ddH_2_O, 800 μL of chloroform (Sigma-Aldrich), and 400 μL of methanol (Sigma-Aldrich). Samples were freeze-thawed three times in liquid nitrogen, and the supernatant was dried using a nitrogen stream. The retained metabolites were then incubated with 120 μL ddH_2_O, and determined by using a SpectraMax iD3 Reader (Molecular Devices, CA, USA) according to the manufacturer’s instructions.

For measurement of the α-KG levels in the cytosol and the nucleus, nucleus or cytosol fractions of tumor cells were prepared using a differential centrifugation method^43,44^. Briefly, at least 1.5 × 10^7^ cells were harvested by a cell scraper, pelleted at 500× *g* for 3 min and washed twice with PBS. The cell pellets were placed on ice and broken by adding 2 mL of hypotonic homogenization buffer (IB 0.1×: 3.5 mM Tris-HCl, pH 7.8, 2.5 mM NaCl, 0.5 mM MgCl_2_) and homogenized using a homogenizer (Wheaton, NJ, USA). Then, 200 µL of hypertonic buffer (IB 10×: 0.35 M Tris-HCl, pH 7.8, 0.25 M NaCl, 50 mM MgCl_2_) was added to make the medium isotonic. This homogenate was centrifuged at 1200× *g* for 3 min at 4 °C to pellet nuclei. The mitochondria contained in the supernatant were pelleted by centrifugation in a microfuge at 15,000× *g* for 2 min at 4 °C, and the supernatant was collected in Eppendorf tubes to measure the α-KG level in the cytosol.

### Glutamine-derived metabolic flux

Steady state metabolic fluxes were performed as previously described^45,46^. For glutamine labeling, cells were cultured with glutamine free DMEM, 12% dialyzed calf serum, and addition of 2 mM ^13^C_5_-Glutamine for 24 h to reach the steady state^47^. After two washes with cold PBS, the cells were incubated with cold methanol (Sigma-Aldrich) at –80 °C for 30 min. Following the addition of 1 mL of cold ddH_2_O, the cells were sent to the Profleader Corporation (Shanghai, China) for metabolic flux analysis. Data were processed by Multiquant 3.0.3 software.

### ACO2 activity assays

ACO2 activity was assayed using a coupled assay following native PAGE separation^48^. Briefly, samples contain cell lysates, 25 mM Tris-HCl (PH 8.0), 10% glycerol, and 0.025% bromophenol blue. Native PAGE gels are composed of a separating gel containing 8% acrylamide, 132 mM Tris-HCl (PH 8.0), 132 mM borate, 3.6 mM citrate, and a stacking gel containing 4% acrylamide, 66 mM Tris-HCl (PH 8.0), 66 mM borate, 3.6 mM citrate. The running buffer contains 25 mM Tris-HCl (PH 8.0), 192 mM glycine, and 3.6 mM citrate. Electrophoresis was carried out at 220 V at 4 °C. Aconitase activities were assayed by incubating the gel in the dark at 37 °C in 100 mM Tris (pH 8.0), 1 mM NADP, 2.5 mM cis-aconitic acid, 5 mM MgCl_2_, 1.2 mM MTT, 0.3 mM phenazine methosulfate, and 5 U/mL isocitrate dehydrogenase.

### Immunoprecipitation and immunoblotting analysis

The extraction of proteins from cultured cells was performed with a modified buffer and was followed by immunoprecipitation and immunoblotting using corresponding antibodies as described previously^49^.

### RT and PCR analysis

Total RNA isolation, RT, and real-time PCR were conducted as described previously^50^. Primer sequences are listed in Supplementary Table S3.

### ChIP-PCR assay

According to the manufacturer’s suggestion (Cell Signaling Technology), for crosslinking of proteins to DNA, PaTu-8988t cells were first fixed with 1% formaldehyde (Sigma-Aldrich) and then neutralized with 1 mL of 10× glycine (Sigma-Aldrich), followed by a cold PBS wash. The cells were then harvested in 1 mL of PBS containing 5 μL of 200× Protease Inhibitor Cocktail (PIC) and centrifuged at 2000× *g* for 5 min at 4 °C. The cell pellet was then resuspended in 1 mL of lysis buffer (1× Buffer A, 0.5 μL of 1 M DTT, 5 μL of 200× PIC) and incubated for 10 min on ice. The pellets were then resuspended in 100 µL of buffer B containing 0.5 μL of micrococcal nuclease to digest DNA, and the mixtures were neutralized with 0.05 M EDTA. After resuspension in 100 μL of ChIP buffer, chromatin was sheared with an ultrasonic homogenizer (Scientz-IID, Ningbo Scientz Biotechnology, Ningbo, China) for five 20” bursts at 30” intervals on ice. Next, 400 μL of 1× Chip buffer was added to the digested chromatin, and 10 μL of the sample was retained as 2% input. The samples were then incubated for 16 h at 4 °C with mouse IgG (Cell Signaling Technology) or anti-c-Myc antibodies (Cell Signaling Technology) according to the manufacturer’s suggestion. The next day, 30 μL of protein G agarose beads was added to each sample, and the immunoprecipitation mixture was washed with low- and high-salt washes. Chromatin was eluted overnight, and the following day, DNA was purified using spin columns, and real-time PCR was performed as described above. Primer sequences are listed in Supplementary Table S3.

### Luciferase reporter assay

C-Myc activity was measured by a luciferase assay system as previously described^51^. In brief, a 1431 bp DNA fragment, predicted to contain the transcription factor binding site upstream of the *Iscu2* gene, was synthesized by Tsingke Biological Technology (Hangzhou, China) and cloned into the pLV(Exp) plasmid (Promega, WI, USA). pLV(Exp), pRL-TK (as an inner control that contains Renilla luciferase sequences, Promega), and c-Myc siRNA or expression vector containing c-Myc were cotransfected into HEK293T cells. Following transfection for 24 h, the cells were washed with PBS and lysed with 250 μL of fresh passive lysis buffer (Luciferase Assay Reagent, Promega, WI, USA) for 15 min^52^. Following the addition of 100 μL of Luciferase Assay Reagent II (Luciferase Assay Reagent, Promega) to 96-well flat-bottom white plates, 20 μL of the cell lysate was transferred to the plates. Firefly luciferase activity was monitored using a SpectraMax iD3 Reader (Molecular Devices) and normalized against *Renilla* luciferase.

### Cell proliferation

Approximately 1.5 × 10^4^ cells were plated in triplicate in 12-well plates and cultured for the appropriate time until they were ready for counting. The cells were then collected for cell counting using a NovoCyte flow cytometer (NovoCyte 2040 R, Agilent Technologies, Inc., CA, USA). In addition to genetic interference, dimethyl α-KG, L-glutamine, or octyl-(R)-2HG (Sigma-Aldrich) was added to evaluate their contribution to cell proliferation.

### Fe–S cluster fuorescent assay

Fe–S cluster fuorescent assay was performed as described^23^. Fe–S fuorescent sensor plasmids were generated by cloning genes encoding N173 (Venus’s residues 1–173) or C155 (Venus’s residues 155–243) fused to GRX2 through a 15 amino acid (GGGGS)_3_ linker into pCDNA3.4. N173- and C155-terminal Venus-GRX2 fusion constructs were mixed in 1:1 v/v ratio and co-transfected into PANC-1 cells with or without KRAS depletion by siRNA. After 72 h, fluorescence was quantified by flow cytometry.

### OCR assay

The OCR assays were performed, as described previously^53^, using an Oxygraph‐2k (Oroboros, Innsbruck, Austria). Briefly, about 1.5 × 10^6^ cells were harvested and added to the chamber. After recording the basal respiration, oligomycin (2.5 μg/mL; Sigma‐Aldrich) was added to the chamber to determine the proton leaking respiration. To determine the maximum OCR, we added cyanide 4-(trifluoromethoxy) phenylhydrazone (FCCP, 2 mM, Sigma-Aldrich). All respiratory data were normalized to the cell number.

### Analysis of apoptosis

The cell apoptosis rate was measured by using a propidium iodide (PI)/Annexin V apoptosis detection kit (BD Bioscience, NJ, USA), as previously described^54^. Briefly, cells were plated in 12-well plates. After the indicated treatment, all cells, including those floating in the medium, were harvested (approximately 1 × 10^5^ cells) and incubated with 5 μM PI and 5 μM Annexin V (BD Bioscience, NJ, USA) for 15 min in the dark. The cells were then analyzed using a NovoCyte flow cytometer (Agilent).

### Cell cycle assay

Cell cycle analyses were performed as previously described^55^. Approximately 1 × 10^5^ cells were collected and centrifuged, and the pellets were added dropwise to 5 mL of cold 70%–80% ethanol. Following incubation at 4 °C for 14–16 h, the cells were washed twice to remove ethanol (first wash in 1× PBS and the second in Stain Buffer). Cell pellets were then incubated with 0.5 mL of PI/RNase Staining Buffer (BD Bioscience) for 15 min in the dark and analyzed using a NovoCyte flow cytometer (Agilent).

### Tumorsphere-forming assay

Tumorsphere-forming assays were performed as previously described^56,57^. Briefly, 5 × 10^3^ cells/well were seeded in triplicate in 6-well plates, and the cells were cultured in DMEM/Ham’s F12 (1:1) medium (1× B27, N2, 20 ng/mL epidermal growth factor (EGF), 10 ng/mL basic fibroblast growth factor (bFGF); Sigma-Aldrich). After 5 days, tumorspheres with diameters exceeding 50 μm were counted under a light microscope (Nikon Corporation, Tokyo, Japan).

### Colony formation assay

Cells (1 × 10^3^ cells/well) were plated in triplicate in 6-well plates (Corning) at a density of 500 cells/well. The cells were cultured for 15 days, the medium was discarded, and 500 μL of 4% paraformaldehyde (Shanghai Lingfeng Chemical Reagent Co., Ltd., Shanghai, China) was added to the plates for 20–30 min. Colonies were then stained with 0.1% crystal violet (Beyotime) for 20–30 min, and colonies whose diameters exceeded 0.5 mm were counted under a light microscope^58^.

### Immunofluorescence analysis

Immunofluorescence analysis was performed according to standard protocols^59^. Cells (1.5 × 10^4^) were seeded in triplicate on coverslips (WHB, Shanghai, China) in 24-well plates. After 48 h, the cells were fixed with 70% ethanol and permeabilized with 1.5 M HCl (Lanxi Zhongxing Chemical Reagent Co., Ltd., Lanxi, China). The cells were then washed twice with PBS and blocked with 5% goat serum (Absin Bioscience, Inc., Shanghai, China) and 0.3% Triton^TM^ X-100 (Sigma-Aldrich) in 1× PBS. Next, the cells were stained with primary antibodies against 5mC (#28692, 1: 1600, Cell Signaling Technology) or at 4 °C overnight^34^. The next day, the cells were incubated with anti-rabbit IgG-Alexa Fluor^TM^ 488 antibody (#8878; 1:500, Cell Signaling Technology) in the dark on a gyratory shaker (Kylin-Bell Lab Instruments, Haimen, Jiangsu, China). Coverslips were then mounted with 5 μL of mounting medium containing DAPI (Beyotime). At least 100 cells from each biological replicate were captured at 600× magnification using a confocal laser-scanning microscope (Nikon Corporation). The fluorescence signal intensity was measured using ImageJ v. 2.4.1.7 (National Institutes of Health, MD, USA).

### IHC analysis and scoring

As described previously^60^, sections of paraffin-embedded tissue were stained with an anti-IscU antibody for human PDAC tissues, with anti-5mc antibody for xenograft tissues, and with nonspecific IgG as a negative control. The paraffin was removed using xylene (Changshu Yangyuan Chemical Co., Ltd., Changshu, China), and the sections were rehydrated in ethanol solutions of different concentrations. The activity of the antigen was restored by incubating sections in 10 mM citric acid for 5 min; this step was repeated four times. A drop of H_2_O_2_ was added to block the activity of endogenous peroxide enzymes. The sections were then incubated with primary anti-IscU and visualized using diaminobenzidine (DAB; Beyotime). The sections were then dehydrated with different concentrations of ethanol solution, clarified with xylene, and sealed with neutral resin. Protein expression was observed under a light microscope, and the integrated optical density (IOD) was calculated using Image-Pro Plus 6.0 (Media Cybernetics, MD, USA). Finally, the mean optimal density (MOD), which represents the IscU intensity, was calculated as IOD/area.

### Animal experiments

As described previously^61^, the mice (6 weeks old) were injected subcutaneously with 5 × 10^6^ PaTu-8988t cells or PANC-1 cells under the armpit or in the shoulder, and the cells were resuspended in a mixture of PBS and Matrigel (Corning, Corning, NY, USA) at a ratio of 1:1. When the tumors were established, their sizes were measured using a Vernier caliper once or twice a week. Tumor volume was calculated according to the following formula: volume = (length × width × height) × 0.5236. For mice intraperitoneally injected with α-KG, when the tumors were established, the mice were randomly divided into two groups and intraperitoneally injected with dimethyl α-KG (DM-α-KG) or physiological saline solution daily (600 mg/kg)^16^. After approximately one month, all mice were sacrificed and carefully dissected to obtain the tumors, which were photographed and weighed. The tumor tissue was harvested, fixed in 4% formaldehyde, and embedded in paraffin.

### WGBS, ox-WGBS, and RNA sequencing

All sequencing experiments were performed using Illumina instruments (Illumina, Inc., USA) at the Beijing Genomics Institute (Beijing, China). For evaluation of the differentially methylated cytosines (DmCs) and differentially hydroxymethylated cytosines (DhmCs) compared with the control cells, a WGBS library and ox-WGBS library were prepared to quantify 5mC and 5hmC levels as described previously^62^. After low-quality bases and adapter sequences were trimmed, clean data were mapped to the human genome (hg38) using BSMAP software (version 2.73). Only cytosines in a CpG context with sufficient sequencing depth (greater than or equal to 5× coverage) were retained for DmC or DhmC detection by SMART (version 2.2.8). DmCs or DhmCs were considered significant with a differential methylation rate between the cells transfected with *IscU2* siRNA and control cells of no less than 0.2 and a *P* value < 0.05. Metilene (version 0.23) was used to define the differentially methylated regions (DMRs) and differentially hydroxymethylated regions (DhMRs) when the methylation/hydromethylation difference was greater than 0.1, and the CpG number contained in DMRs/DhMRs was greater than 5. For the integration of transcriptome data, only methylation/hydromethylation in the promoter regions (2 kb upstream and 0.5 kb downstream) was used for subsequent analysis. For transcriptome analysis, total RNA was extracted from the cells transfected with *IscU2* siRNA and control cells for sequencing on an Illumina instrument. Data were cleaned as described above and mapped to hg38 using Bowtie2 software (version 2.3.4.2). Next, the gene counts were analyzed and normalized using the DESeq2 package (version 1.24.0) to identify DEGs. With the Benjamin–Hochberg method, a *P* value cutoff of 0.05 was used. The biological functions of DEGs were assigned manually according to the published literature and the Gene Ontology database (http://geneontology.org/). A bar plot was drawn using the ggplot2 package in R software (version 3.3.2).

### TCGA and GTEx data analysis

The combined gene counts of TCGA and GTEx datasets were downloaded from the UCSC Xena browser website (https://xenabrowser.net/), and data containing 178 PDAC and 171 normal pancreatic samples were extracted using custom Perl scripts (Strawberry Perl 5.28.1.1) for subsequent analysis. The DESeq2 R package (version 1.24.0) was used for differential expression analysis and gene expression normalization. Statistical significance was set at *P* < 0.05. A heatmap was generated using the pheatmap package in R software (version 1.0.12).

### Statistical analysis

All quantitative experiments were performed using at least three biological replicates. Data are presented as means ± SEM. Statistical analyses were performed by either paired or unpaired two-tailed Student’s *t*-test for two-group comparisons using SPSS (version 22.0; IBM, NY, USA), unless otherwise specified in the figure legends. Values of *P* < 0.05 were considered significant.

## Supplementary information

Supplementary Figures

Supplementary tables

Supplementary dataset
