# Supplementary material for: The Fe–S cluster assembly protein IscU2 increases α-ketoglutarate catabolism and DNA 5mC to promote tumor growth
Source: Cell Discov. 2023 Jul 25;9:76. doi: 10.1038/s41421-023-00558-8 (PMC10366194; doi:10.1038/s41421-023-00558-8)
Supplement: Supplementary file 1 — Supplementary Figures [file 41421_2023_558_MOESM1_ESM.pdf]

Supplementary Figure 1

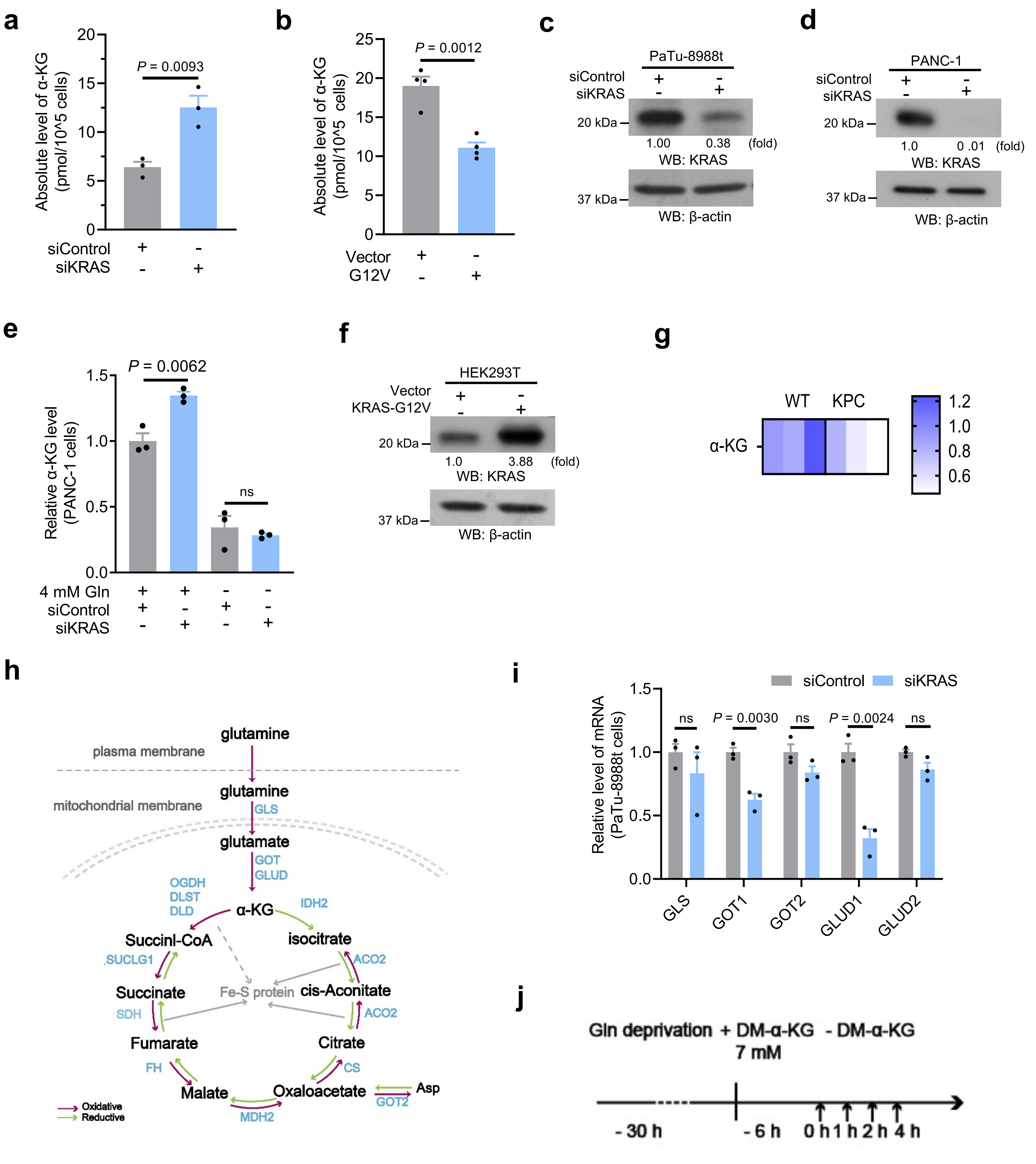

Supplementary Figure 2

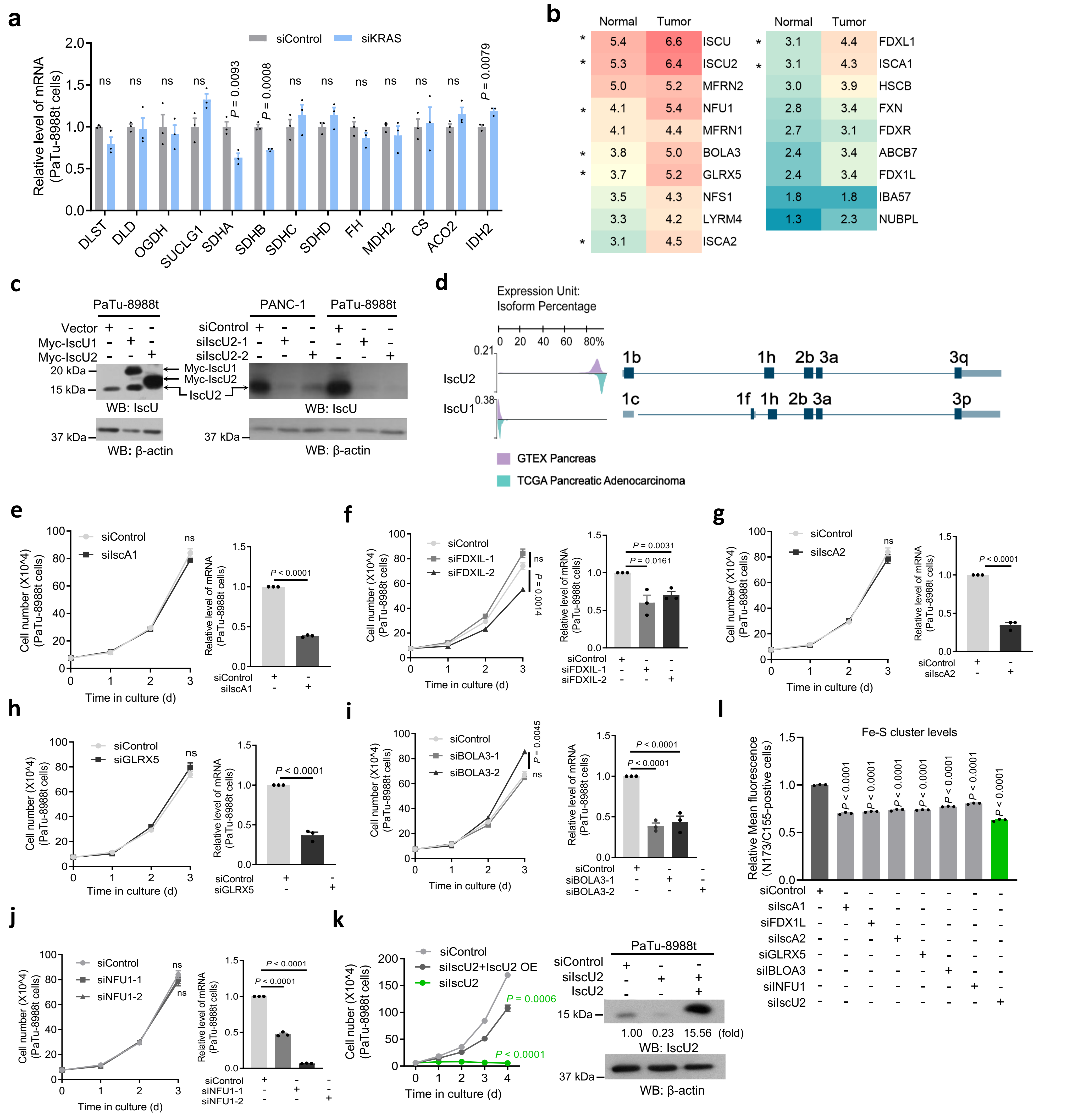

Supplementary Figure 3

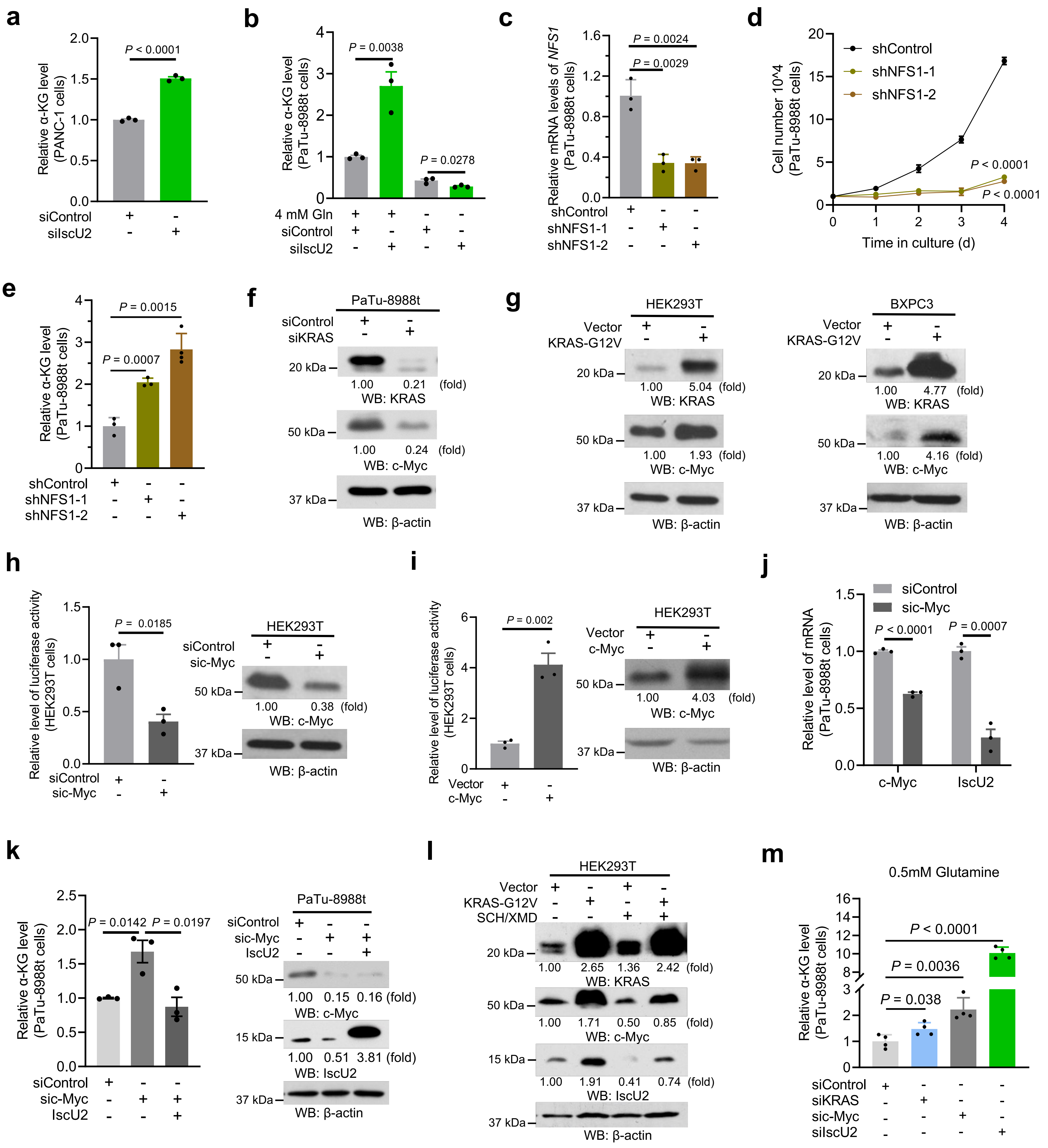

Supplementary Figure 4

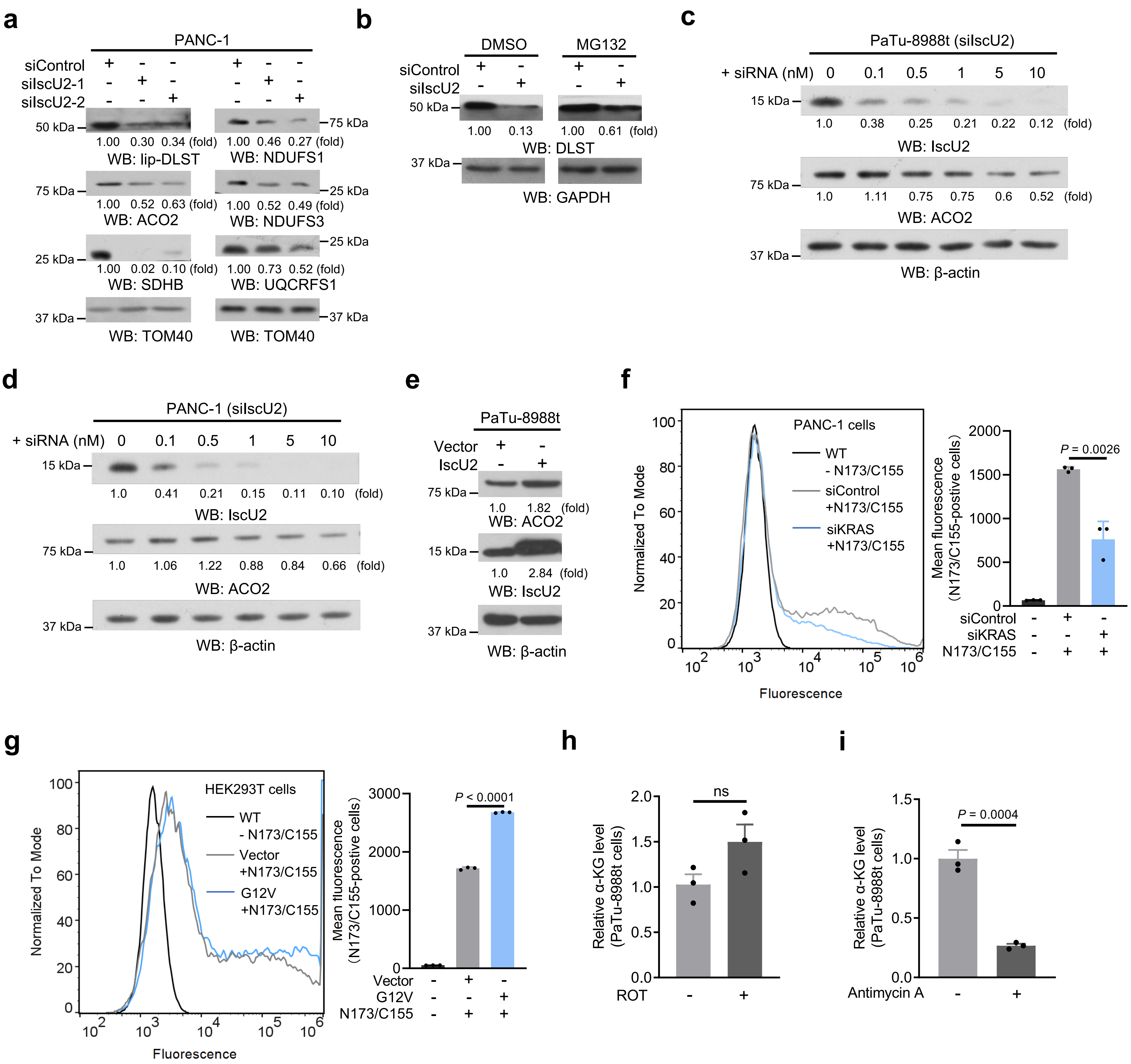

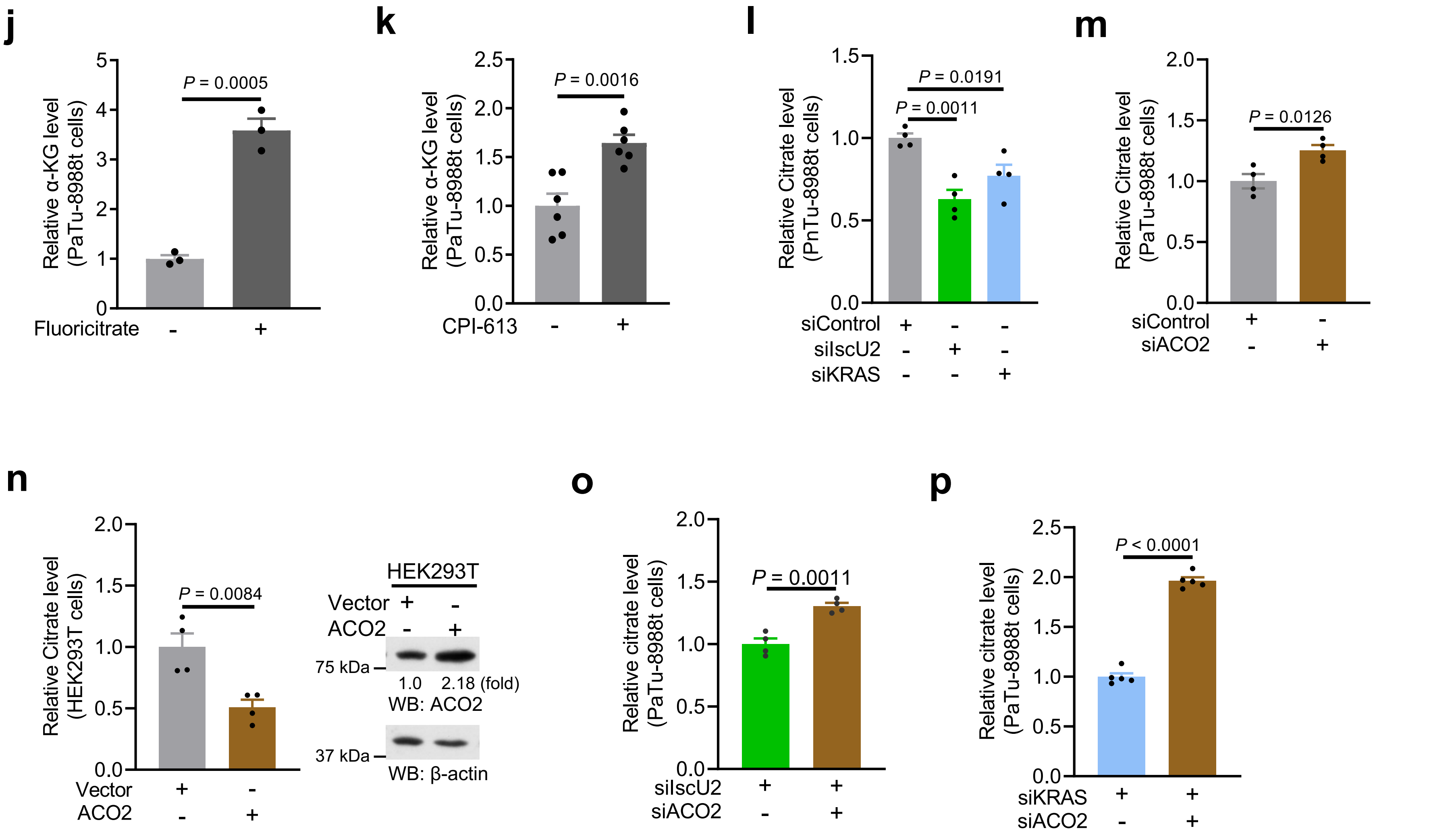

j
Relative α-KG level (PaTu-8988t cells)
P = 0.0005
Fluoricitrate - +
k
Relative α-KG level (PaTu-8988t cells)
P = 0.0016
CPI-613 - +
l
Relative Citrate level (PnTu-8988t cells)
P = 0.0191
P = 0.0011
siControl + - -
siIscU2 - + -
siKRAS - - +
m
Relative Citrate level (PaTu-8988t cells)
P = 0.0126
siControl + -
siACO2 - +
n
Relative Citrate level (HEK293T cells)
P = 0.0084
Vector + -
ACO2 - +
HEK293T
Vector + -
ACO2 - +
75 kDa
1.0 2.18 (fold)
WB: ACO2
37 kDa
WB: β-actin
o
Relative citrate level (PaTu-8988t cells)
P = 0.0011
siIscU2 + +
siACO2 - +
p
Relative citrate level (PaTu-8988t cells)
P < 0.0001
siKRAS + +
siACO2 - +

Supplementary Figure 5: related to figure 4

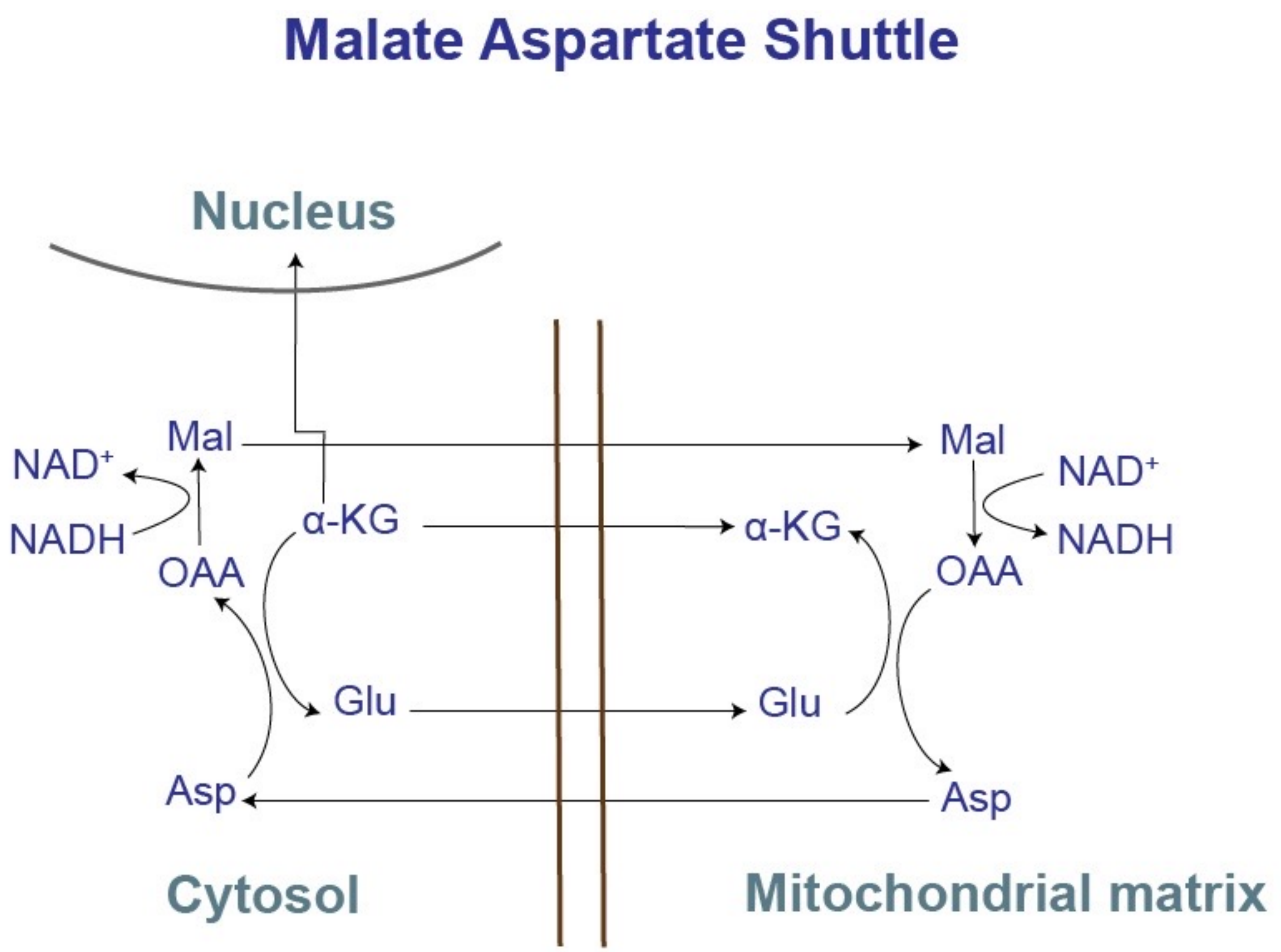

Mal:Malate; OAA: Oxaloacetate; Glu: Glutamate; Asp: Aspartate; α-KG: α-ketoglutarate

Supplementary Figure 6

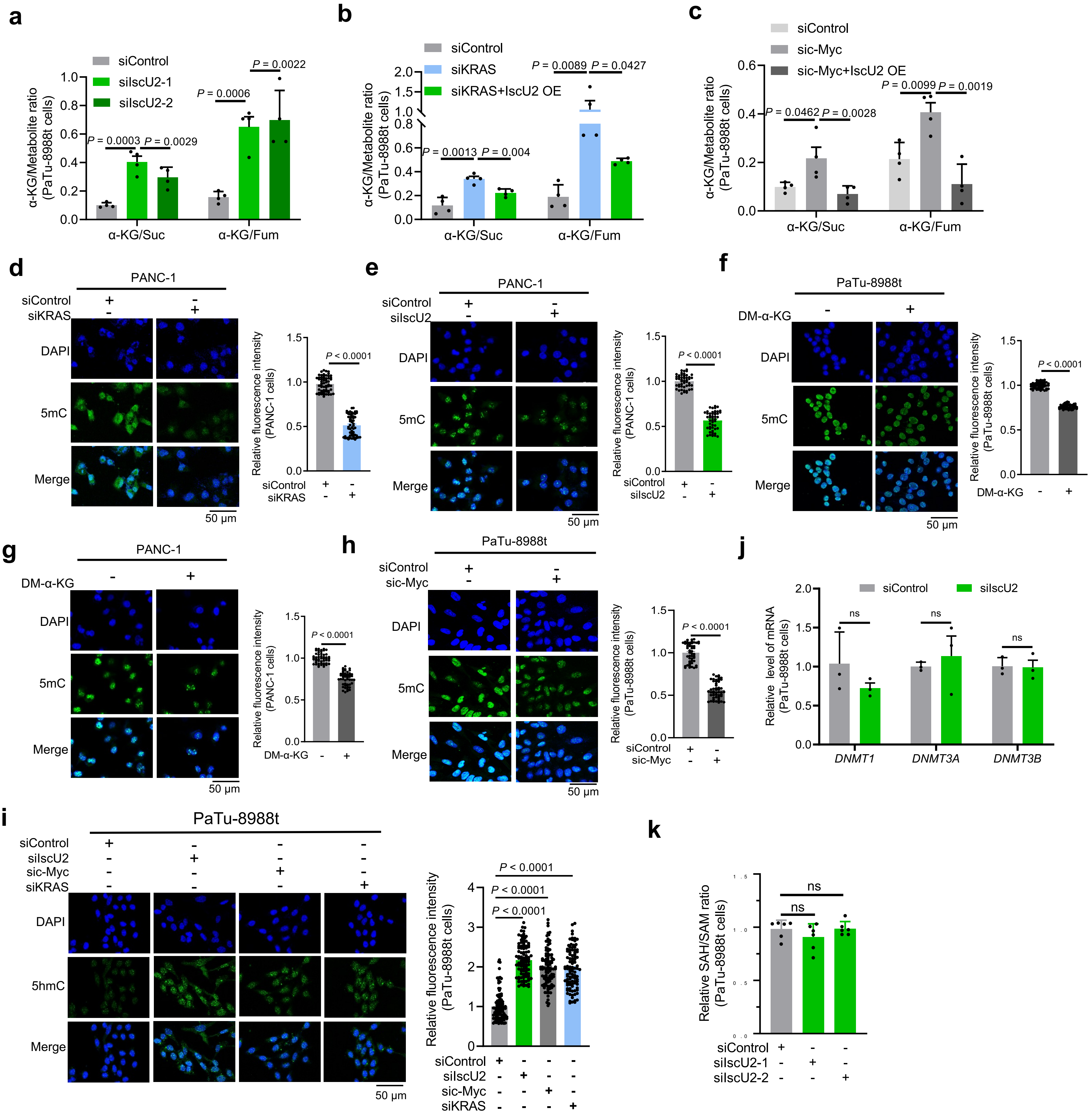

l

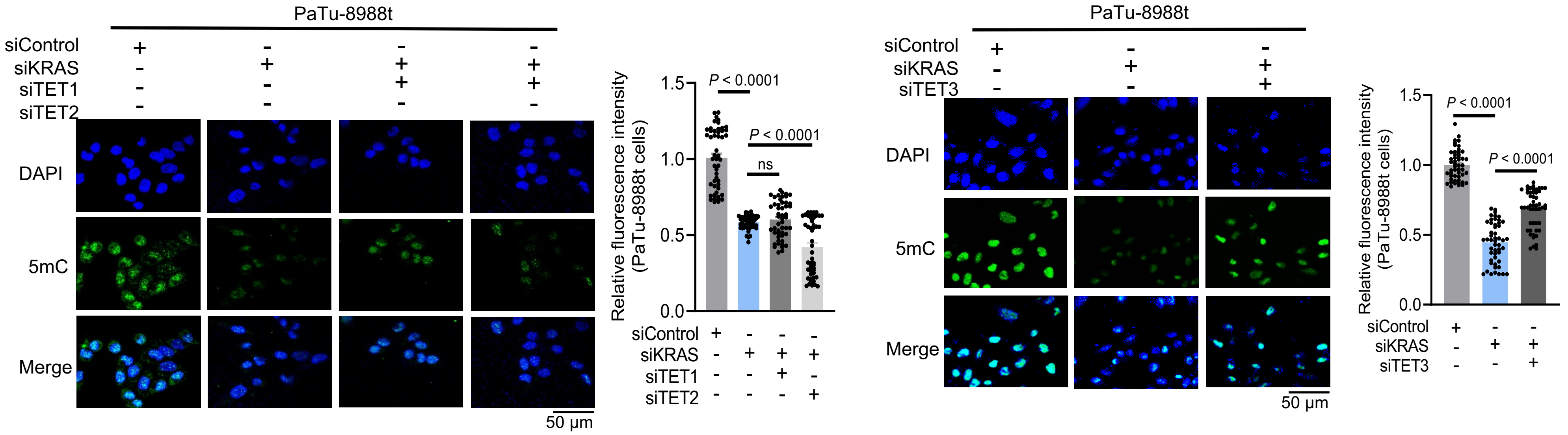
PaTu-8988t
siControl
siKRAS
siTET1
siTET2
DAPI
5mC
Merge
50 μm
Relative fluorescence intensity (PaTu-8988t cells)
P < 0.0001
ns
siTET3

m

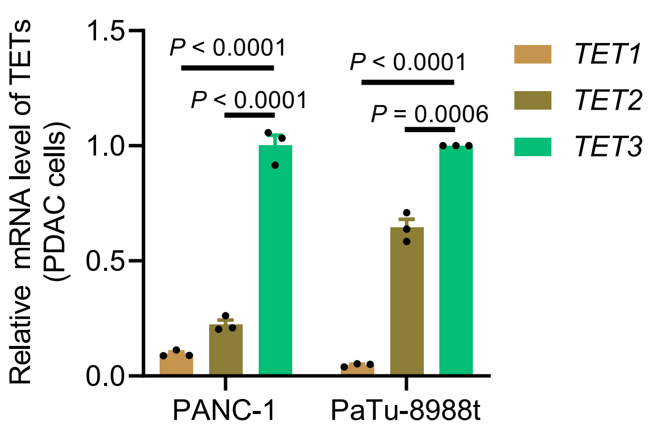
Relative mRNA level of TETs (PDAC cells)
P < 0.0001
P = 0.0006
TET1
TET2
TET3
PANC-1
PaTu-8988t

n

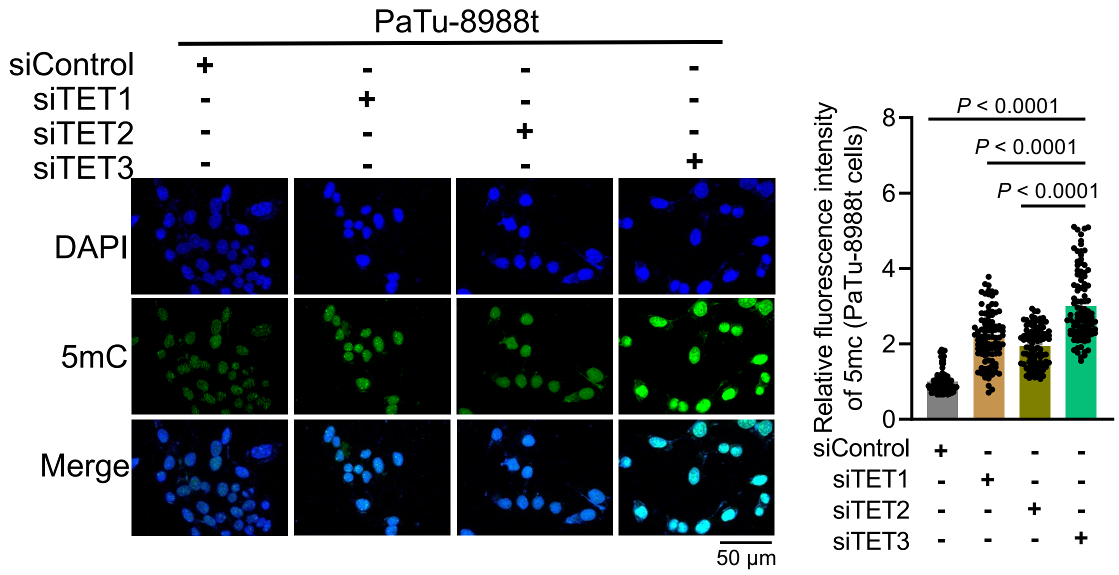
PaTu-8988t
siControl
siTET1
siTET2
siTET3
DAPI
5mC
Merge
50 μm
Relative fluorescence intensity of 5mc (PaTu-8988t cells)
P < 0.0001

o

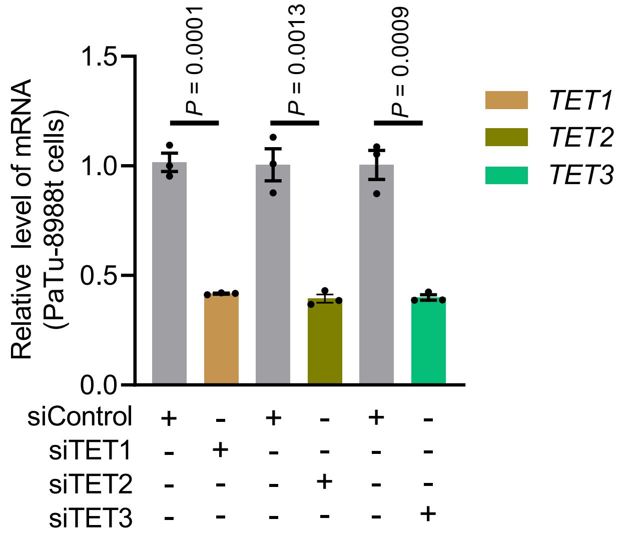
Relative level of mRNA (PaTu-8988t cells)
P = 0.0001
P = 0.0013
P = 0.0009
TET1
TET2
TET3
siControl
siTET1
siTET2
siTET3

p

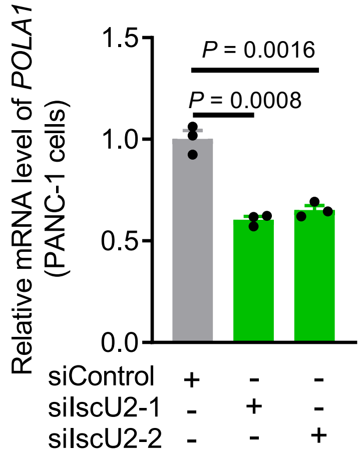
Relative mRNA level of POLA1 (PANC-1 cells)
P = 0.0016
P = 0.0008
siControl
siIscU2-1
siIscU2-2

Supplementary Figure 7

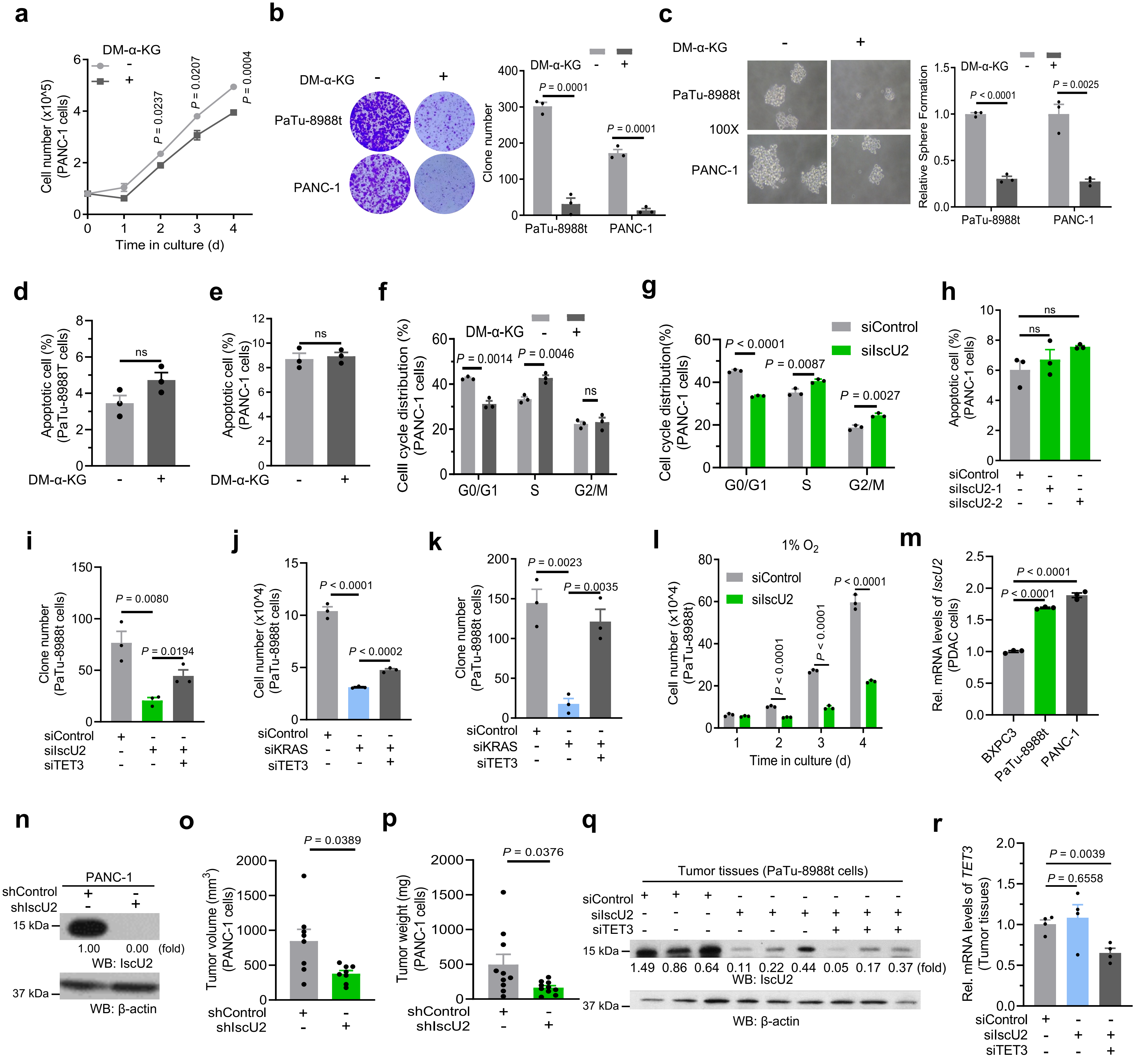

Supplementary Fig. S1. KRAS increases the rates of α-KG catabolism in PDAC cells.

a Absolute α-KG levels of PaTu-8988t cells transfected with a control siRNA or KRAS siRNA (means ± SEM, *n* = 3).

b Absolute α-KG levels of HEK293T cells expressed empty vector or KRAS G12V mutant (means ± SEM, *n* = 4).

c, d PaTu-8988t and PANC-1 cells were transfected with a control siRNA or KRAS siRNA, immunoblot analyses were performed with the indicated antibodies.

e Relative α-KG level of PANC-1 cells cultured in medium with or without 4 mM glutamine (Gln) for 24 h were determined and normalized to the number of cells (means ± SEM, *n* = 3).

f HEK293T cells expressed empty vector or KRAS G12V mutant. Immunoblot analyses were performed with the indicated antibodies.

g Analysis of the α-KG levels in PDAC tissues from the KRAS (G12D)/Trp53 null/Pdx1-cre (KPC) mice and normal pancreases from the WT mice. Related metabolic raw data were downloaded from the published literature [20].

h Schematic of the enzymes involved in glutaminolysis and the TCA cycle. The black arrows indicate Fe-S proteins; the dotted arrow indicated enzymes whose functions are indirectly dependent on Fe-S cluster.

i qPCR analysis of GLS, GOT1, GOT2, GLUD1, and GLUD2 in PaTu-8988t cells with or without transfection with KRAS siRNA (means ± SEM, *n* = 3).

j Schematic diagram for a time-course experiment to determine the α-KG levels. The PaTu-8988t cells were pretreated with medium lacking glutamine for 24 h (-30 h to -6 h) and subjected to culture in medium with DM-α-KG (7 mM) for an additional 6 h (-6 h to 0 h). The α-KG levels in the PaTu-8988t cells with or without transfection of KRAS siRNA were measured at -6 h, 0 h, 1 h, 2 h, and 4 h.

Statistical significance was determined by unpaired two-tailed Student's *t* test, ns not significant.

Supplementary Fig. S2. IscU2 expression is much higher in the human PDAC tissues and IscU2 promotes PDAC cell proliferation.

a PaTu-8988t cells were transfected with a control siRNA or KRAS siRNA. qPCR analyses were performed with primers targeting the indicated genes. Data are presented as the means ± SEM from three independent experiments.

b mRNA levels of the component genes of the iron-sulfur cluster assembly machinery in normal (GTEx, n = 171) and PDAC tissues (TCGA, n = 179). *, statistically significant, analyzed by one-way ANOVA.

c PANC-1 cells and PaTu-8988t cells were transfected with a control siRNA or IscU1/2 siRNA. IscU1 or IscU2 was stably overexpressed in PaTu-8988t cells. Immunoblot analyses were performed with the indicated antibodies.

d Schematic diagram of the IscU1 and IscU2 levels in normal pancreatic and PDAC tissues (GTEx and TCGA databases). This image was generated by UCSC (http://xena.ucsc.edu/welcome-to-ucsc-xena).

e-j PaTu-8988t cells were transfected with a control siRNA or siRNAs for the indicated genes. Cell proliferation was examined for the indicated cells (means ± SEM, $n = 3$).

k PaTu-8988t cells were transfected with a control siRNA, IscU2 siRNA, or vectors expressing IscU2 siRNA (targeting the noncoding region) and RNAi-resistant IscU2. Cell proliferation was examined (means ± SEM, $n = 3$)， immunoblot analyses were performed with the indicated antibodies.

l Fluorescence of GRX2-Fe-S dependent sensors in PaTu-8988t cells transfected with indicated siRNA (means ± SEM, $n = 3$).

Statistical significance was determined by unpaired two-tailed Student’s t test, ns not significant.

Supplementary Fig. S3. IscU2 expression upregulated by c-Myc promotes α-KG catabolism.

a Relative α-KG levels in PANC-1 cells transfected with a control siRNA or IscU2 siRNA (means ± SEM, $n = 3$).

b PaTu-8988t cells were transfected with a control siRNA or IscU2 siRNA and cultured in medium with or without 4 mM glutamine (Gln) for 24 h. The α-KG levels were determined and normalized to the number of cells (means ± SEM, $n = 3$).

c Relative *NFS1* mRNA levels of PaTu-8988t cells with or without NFS1 depletion by shRNA (means ± SEM, $n = 3$).

d Cell growth of PaTu-8988t cells with or without NFS1 depletion by shRNA (means ± SEM, $n = 3$).

e Relative α-KG levels of PaTu-8988t cells with or without NFS1 depletion by shRNA (means ± SEM, $n \geq 3$).

f, g PaTu-8988t cells were transfected with a control siRNA or KRAS siRNA. Human KRAS G12V was expressed in HEK293T cells and BXPC3 cells. Immunoblot analyses were performed with the indicated antibodies.

h, i HEK293T cells were transfected with the luciferase reporter vector containing the sequence of the *IscU2* promoter (-1500 bp to the transcription start site). After transfection with a control siRNA, c-Myc siRNA, or a vector expressing c-Myc for 24 h, luciferase activity was measured (means ± SEM, n = 3). Immunoblot analyses were performed with the indicated antibodies.

j PaTu-8988t cells were transfected with a control siRNA or c-Myc siRNA. qPCR analyses were performed with primers targeting the indicated genes (mean ± SEM, n = 3).

k Relative α-KG levels in PaTu-8988t cells transfected with a control siRNA, c-Myc siRNA, or c-Myc siRNA with a vector expressing IscU2 (means ± SEM, n = 3). Immunoblot analyses were performed with the indicated antibodies.

l HEK293T cells expressed empty vector or KRAS G12V were treated with or without 2 μM SCH772984 and XMD8-92. Immunoblot analyses were performed with the indicated antibodies.

m PaTu-8988t cells were transfected with a control siRNA, KRAS siRNA, c-Myc siRNA or IscU2 siRNA and cultured in 0.5 mM Gln medium for 24 h. Relative α-KG levels were determined (means ± SEM, n = 4).

Statistical significance was determined by unpaired two-tailed Student's t test, ns not significant.

Supplementary Fig. S4. The role of activated KRAS in promoting α-KG catabolism relied on IscU2-mediated Fe-S clusters assembly function.

a PANC-1 cells were transfected with a control siRNA or IscU2 siRNA. Immunoblot analyses were performed with the indicated antibodies.

b PaTu-8988t cells transfected with a control siRNA or IscU2 siRNA were pretreated with or without MG132 (10 mM) for 12 h. Immunoblot analyses were performed with the indicated antibodies.

c-d Immunoblot analyses were performed with the indicated antibodies. PaTu-8988t cells (**c**) and PANC-1 (**d**) were transfected with different concentrations of IscU2 siRNA.

e Immunoblot analysis of indicated proteins in PaTu-8988t cells stably overexpressed empty control vector or IscU2.

f Fluorescence of GRX2-Fe-S dependent sensors in PANC-1 cells transfected with a control siRNA or KRAS siRNA (means ± SEM, n = 3).

g Fluorescence of GRX2-Fe-S dependent sensors in HEK293T cells expressed empty vector or KRAS G12V mutant (means ± SEM, n = 3).

h-k PaTu-8988t cells were treated with or without rotenone (50 nM) for 24 h (**h**), antimycin A (100 nM) for 24 h (**i**), fluorocitrate (50 μM) for 24 h (j), or CPI-613 (240 μM) for 2 h (**k**). α-KG levels were determined and normalized to the number of cells (means ± SEM, $n \geq 3$).

l Relative citrate levels in PaTu-8988t cells transfected with a control siRNA, IscU2 siRNA or KRAS siRNA (means ± SEM, n = 4).

m Relative citrate levels in PaTu-8988t cells transfected with a control siRNA or ACO2 siRNA (means ± SEM, n = 4).

n Relative citrate levels in PaTu-8988t cells with or without overexpression of ACO2 (means ± SEM, n = 4). Immunoblot analyses were performed with the indicated antibodies.

o, p Relative citrate levels in PaTu-8988t cells transfected with indicated siRNA (means ± SEM, $n \geq 4$).

Statistical significance was determined by unpaired two-tailed Student's *t* test, ns not significant.

Supplementary Fig. S5. Schematic diagram of malate-aspartate shuttle.

Supplementary Fig. S6. KRAS promotes DNA 5mC in PDAC cells through IscU2-enhanced α-KG catabolism and subsequent TET3 inhibition.

a The ratios of α-KG/succinate and α-KG/fumarate of PaTu-8988t cells transfected with a control siRNA or IscU2 siRNAs (means ± SEM, n = 4).

b The ratios of α-KG/succinate and α-KG/fumarate of PaTu-8988t cells transfected with a control siRNA, KRAS siRNA, or KRAS siRNA with a vector expressing IscU2 (means ± SEM, n = 4).

c The ratios of α-KG/succinate and α-KG/fumarate of PaTu-8988t cells transfected with a control siRNA, c-Myc siRNA, or c-Myc siRNA with a vector expressing IscU2 (means ± SEM, n = 4).

d-h PANC-1 cells were transfected with a control siRNA, IscU2 siRNA or KRAS siRNA (**d** and **e**). PaTu-8988t cells and PANC-1 cells were pretreated with or without DM-α-KG (7 mM)

for 48 h (**f** and **g**). PaTu-8988t cells were transfected with a control siRNA or c-Myc siRNA **h** Immunofluorescence analyses were performed with the indicated antibody.

i PaTu-8988t cells were transfected with a control siRNA, IscU2 siRNA, c-Myc siRNA, or KRAS siRNA. Immunofluorescence analyses were performed with the indicated antibody.

j qPCR analysis of *DNMT1*, *DNMT3A*, and *DNMT3B* in PaTu-8988t cells transfected with a control siRNA or IscU2 siRNA (means ± SEM, n = 3).

k Relative levels of SAM and SAH in PaTu-8988t cells transfected with a control siRNA, IscU2 siRNAs (means ± SEM, n = 6).

l PaTu-8988t cells were transfected with a control siRNA, KRAS siRNA, or co-transfected with KRAS siRNA and TET1/2/3 siRNAs. Immunofluorescence analyses were performed with the indicated antibody.

m qPCR analysis of the relative mRNA levels of *TET1, TET2* and *TET3* in PANC-1and PaTu-8988t cells.

n Immunofluorescence analyses of PaTu-8988t cells transfected with a control siRNA, TET1 siRNA, TET2 siRNA or TET3 siRNA. Nuclei were stained with DAPI. Quantification performed from 3 experiments with 50 cells quantified for each condition. Scale bars represent a distance of 50 μm.

o qPCR analysis of *TET1, TET2* and *TET3* in PaTu-8988t cells transfected with a control siRNA, TET1 siRNA, TET2 siRNA or TET3 siRNA (means ± SEM, n = 3).

p qPCR analysis of *POLA1* in PANC-1 cells transfected with a control siRNA or IscU2 siRNA (means ± SEM, n = 3).

Statistical significance was determined by unpaired two-tailed Student’s *t* test, ns not significant.

Supplementary Fig. S7. IscU2 expression upregulated by activated KRAS promotes DNA 5mC-dependent PDAC cell proliferation and tumor growth in mice.

a-f PANC-1 or PaTu-8988t cells were treated with or without DM-α-KG (7 mM). Cell proliferation (**a**), plate clone formation (**b**), tumorsphere-forming (**c**), cell apoptosis (**d**, **e**) and cell cycle progression (**f**) were detected (means ± SEM, n = 3).

g, h PANC-1 transfected with a control siRNA or IscU2 siRNA were plated for 48h. Cell cycle progression (**g**), and the percentage of apoptotic cells (**h**) were determined (means ± SEM, n = 3).

i Plate colony formation of PaTu-8988t cells transfected with indicated siRNA was detected (means ± SEM, $n = 3$).

j, k Cell proliferation (**j**) and plate colony formation (**k**) of PaTu-8988t cells transfected with indicated siRNA (means ± SEM, $n = 3$).

l Cell proliferation under hypoxia (1% O_2) of PaTu-8988t transfected with a control siRNA or IscU2 siRNA (means ± SEM, $n = 3$).

m qPCR analysis of *IscU2* in BXPC3, PaTu-8988t, and PANC-1 cells (means ± SEM, $n = 3$).

n-p PANC-1 cells were transfected with a control shRNA or IscU2 shRNA and immunoblot analyses were performed with the indicated antibodies (**n**). PANC-1 cells (5×10^6) with or without expression of the IscU2 shRNA were subcutaneously injected into the flank regions of athymic nude mice. The mice were examined for 7 weeks after injection. The tumor volumes were calculated (o). The tumor weights were measured (**p**). Data are presented as the means ± SEM ($n \geqslant 8$ mice/group).

q Immunoblot analyses of IscU2 in tumor tissues with the indicated antibodies.

r qPCR analysis of *TET3* in tumor tissues (means ± SEM, $n = 4$).

Statistical significance was determined by unpaired two-tailed Student's t test, ns not significant.
