# Supplementary material for: The Fe–S cluster assembly protein IscU2 increases α-ketoglutarate catabolism and DNA 5mC to promote tumor growth
Source: Cell Discov. 2023 Jul 25;9:76. doi: 10.1038/s41421-023-00558-8 (PMC10366194; doi:10.1038/s41421-023-00558-8)
Supplement: Supplementary file 2 — Supplementary tables [file 41421_2023_558_MOESM2_ESM.pdf]

Supplementary Table S1. Transcription factor binding prediction of *IscU2* promoter

**Factor name**	**Start***	**End**	**Predicted sequence**	**Factor name**	**Start**	**End**	**Predicted sequence**
YY1	55	58	CCAT	GR-alpha	233	237	CCTGT
	160	163	CCAT		1174	1178	ATAGG
	271	274	ATGG		1275	1279	CCTGT
	331	334	CCAT	FOXP3	287	292	CACAAC
	345	348	ATGG		719	724	GTTGTT
	383	386	CCAT	GR-beta	213	217	AAATT
	526	529	CCAT		219	223	ACATT
	543	546	CCAT		374	378	AATTT
	768	771	ATGG		649	653	AAATT
	795	798	CCAT		654	658	ACATT
	864	867	ATGG		676	680	AAATT
	1023	1026	CCAT		705	709	AAATT
C/EBP beta	60	63	TTGC	IRF-2	400	405	AAGTGA
	157	160	TTGC	ER-alpha	395	399	GGTCA
	288	291	ACAA		440	444	TGACC
	469	472	GCAA		1365	1369	GGTCA
	486	489	GCAA	STAT4	545	550	ATTTCC
	636	639	GCAA	STAT1 beta	545	554	ATTTCCCAGA
	686	689	TTGC	c-Myc	601	606	CACGTG
	714	717	TTGC	PR B	643	649	AACAGTA
	720	723	TTGT	PR A	643	649	AACAGTA
	841	844	TTGC	AP-2alphaA	855	860	GCAGGC
	886	889	TTGC		1412	1417	GCAGGC
	969	972	GCAA		1508	1513	GCAGGC
	973	976	TTGT	TFII-I	296	301	GGACAG
	989	992	ACAA		809	814	GGAAAG
	1169	1172	GCAA		889	894	CTTTCC
	1231	1234	TTGT	Pax-5	315	321	GGGCAGG
	1304	1307	ACAA		866	872	GGGCAGG
	1418	1421	GCAA		1305	1311	CAAGCCC
	1514	1517	GCAA	Sp1	1477	1486	GCCCCGCCCC
	1522	1525	GCAA	ENKTF-1	1504	1511	TGGCGCAG
XBP-1	71	76	ATGACT	CREB	1430	1438	TGACGTCAG
	341	346	AGTCAT	c-Ets-1	318	324	CAGGAAG
TFIID	141	147	TCAAAAA		1046	1052	CAGGAAG
	225	231	TTTTATA	p53	315	321	GGGCAGG
	254	260	TATAAAA		866	872	GGGCAGG
	617	623	TTTTATA	GR	797	803	ATTTTTG
	723	729	TTTTAGA	NFI/CTF	447	454	GCGCTTGG
	754	760	TTTTCAA	Elk-1	316	324	GGCAGGAAG
	798	804	TTTTTGA	GATA-1	250	255	CAGATA

* The site of 1500bp upstream of transcription start site (TSS) is marked as 0. Transcription factors were predicted by combined use of genome.ucsc.edu and http://alggen.lsi.upc.es.

Supplementary Table S2. Targeted sequences of siRNAs

Genes	Targeted Sequence (5' to 3')	
BOLA3-1	GATGCATGGATTGCGGATA	RiboBio, Guangzhou, Guangdong, China
BOLA3-2	GACCCAAATTCTCAAAGAA	
c-Myc	GAGGAGACATGGTGAACCA	
FDX1L-1	GGTGAACGTGGTGTTCGTA	
FDX1L-2	GAGTGAAGACCACCTGGAT	
GLRX5	GTACCTCAATGGCGAGTTT	
ISCA1	GTCAGAGTATTCATCGAAA	
ISCA2	GATCCGCCTCACAGACAGT	
KRAS	GGACGAATATGATCCAACA	
NFU1-1	CTCCAGGGTTCTTGTACCA	
NFU1-2	TGGCAATGATTAAGGAATT	
TET1	GGACTCAGATGATCTATCA	
TET2	GCAACATAAGCCTCATAAA	
TET3	GTCCTGGGTTCCAAGACAA	
Control siRNA	TTCTCCGAACGTGTCACGT	
ACO2	CCCGCTACTACAAGAAACA	GenePharma, Shanghai, China
DLST	GGTGGTGTATAGGGATTAT	
Control siRNA	ACGTGACACGTTCGGAGAA	
IscU-1	CCTCTCCACTGAAGAGCTATGAGAT	[a] Thermo Fisher Scientific, Waltham, MA, USA
IscU-2	CAGTTTCATTGTTCTGAATCCTGTG	

[a] Sequence of control siRNA for IscU was not listed due to manufacture's policy.

Supplementary Table S3. Sequences of qPCR primers

Gene name	Forward Primer (5' to 3')	Reverse Primer (5' to 3')	Manufacturer
ABCB7	ACATGCTGAACCTGAGTGATG	TCGGATTGAATTCTGGGCTAC	Tsingke Biological Technology (Tsingke Corporation, Hangzhou, China)
ACO2	TGACCATCTGATTGAAGCCC	GGTGAATGATTCCAGATCCAGG	
BOLA3	TTCCACTTCACCATCGGATG	TCATACATCGCCCCACAAC	
CS	CATTGACTCTAACCTGGACTGG	ACTTACATTGCCACCCTCATG	
DLD	GATCCCAGAGGTAGAATTCCAG	CAGCCATTCCTTCAACACAG	
DLST	AAGATGAAGTGGTTTGTGAGATTG	TGAGTGTGAAAAGTGGAGTGC	
FDX1L	ATTCACCCTGCCCAAGATC	CCTGGCTATTCCCTCAATCTG	
FH	GGTGAACTAAAGGTGCCAAATG	CTCGCTTCAAGATGCCAAAAG	
GLRX5	AGAAAGACCAAGACTCCAAGTG	ACCAAAATGATCAGTGCAAGC	
GLS	TTCCAGAAGGCACAGACATG	GGCTCAGTACTCTTTCACCAG	
GLUD1	AGGAATGACACCAGGGTTTG	TCAGACTCACCAACAGCAATAC	
GLUD2	GTATAACCTGGGATTGGACCTG	CGTGAAGAGGTTAGTGAGGAAG	
GOT1	CGGATTCTGACCATGAGATCTG	ACCAGATACTCAACCTGCTTG	
GOT2	GTTTGCCTCTGCCAATCATATG	GAGGGTTGGAATACATGGGAC	
IDH2	AGTCTTCGGGTGGCTTTG	CCTCAATCGTCTTCCCATCAG	
IDH3A	GGAGTCGTGCAGAGTATCAAG	AAAGCCCATCTGACATCCG	
IDH3B	CATGAAGGAGAACAAAGTGGC	ACTTCACATGGACTACGTTGG	
IDH3G	AACCATAACCTGCCACCG	ATGTCTATGTCCTTGTGCCG	
ISCA1	CTTCCTTAGTCCGGGCAAC	GACACCAACTTTTACACCTACATG	
ISCA2	CGAAGGGTCAGAATTCCTCAG	GCCAAGCTATCAGAGTCAACC	
ISCU2	GGGAAGATTGTGGATGCTAGG	GCTCCTTGGCGATATCTGTG	
KRAS	GGAGTACAGTGCAATGAGGG	CCATAGGTACATCTTCAGAGTCC	
LDHA	CGTCAGCAAGAGGGAGAAAG	GCCACGTAGGTCAAGATATCC	
MDH2	AGAAGCATGGAGTGTACAACC	ACCAATGACAGGGACGTTG	
ME1	ATCCTCAAGAATGTCTGCCTG	GCCGTAGTCCAATGTAGAGTG	
ME3	*GACGTGTCTTTGAGAATTGCC*	*GTAGCTGTCCAGTGTAAAGGAG*	
NFU1	TCTTTGCATCTGGCTTACCC	GATTACATCCCCTCCATCTTCC	
OGDH	GACCAAAGCCGAACAGTTTTAC	TGCCATGAGTTGTGTAGGATG	
P1	GAAGACCCGCTGAGGCAGAAG	ATGAGTTTTTATATCTGGTGGGAGC	
P2	CATTTCCCAGATTCCTCCCCTTT	TCTCTCTAAAACAACAGCAAACTC	
P3	TCCAGCACCCAGGACAAGCCCTC	GCCGCCATCTTGCCGGCTTGCGCCTG	
POLA1	ATGGCACCTGTGCACGGCG	TTAGGATTTCACGGCACAACCAGCG	
SDHA	TGGTTGTCTTTGGTCGGG	GCGTTTGGTTTAATTGGAGGG	
SDHB	AGACAAGGCTGGAGACAAAC	CCTTCTCTGCATGATCTTCGG	
SDHC	TGTTGCTGAGACACGTTGG	GAGACAGAGGACGGTTTGAAC	
SDHD	AGCACATACACTTGTCACCG	AATAGTCCATCGCAGAGCAAG	
SUCLA2	CATGTTCTACGGCAGGCTAG	GAGATTCCTTTGCTGTTGCTG	
SUCLG1	TTCAGAGTGCAGGAGTTGTG	ATGTCTACGTGATCCATTCCAC	
ACTIN	ACCTTCTACAATGAGCTGCG	CCTGGATAGCAACGTACATGG	
TET1	TTCGTCACTGCCAACCTTAG	ATGCCTCTTTCACTGGGTG	Fuzhou Bio-gene Biotechnology,Fujian, China
TET2	CACTGCATGTTTGGACTTCTG	TGCTCATCCTCAGGTTTTCC	
TET3	ACCAGATCTGCAAACTGCGA	TGAGCTCTGAGCCTGTCTTG	
